# Introducing a new tool to navigate, understand and use International Codes of Nomenclature

**DOI:** 10.7717/peerj.8127

**Published:** 2019-11-25

**Authors:** Evangelos Vlachos

**Affiliations:** CONICET - Museo Paleontológico Egidio Feruglio, Trelew, Chubut, Argentina

**Keywords:** Nomenclature, Taxonomy, Zoology, Taxon, Code, Species, Network analysis

## Abstract

**Background:**

In order to designate the various concepts of taxa in biology, evolution and paleontology, scientists have developed various rules on how to create unique names for taxa. Different Codes of Nomenclature have been developed for animals, plants, fungi, bacteria etc., with standard sets of Rules that govern the formation, publication and application of the nomina of extant and extinct species. These Codes are the result of decades of discussions, workshops, publications and revisions. The structure and complexity of these Codes have been criticized many times by zoologists. This project aims, using the International Code of Zoological Nomenclature as a case study, to show that the structure of these Codes is better reflected and understood as networks.

**Methods:**

The majority of the text of the Code has been divided into hundreds of Nodes of different types, connected to each other with different types of Edges to form a network. The various mathematical descriptors of the entire system, as well as for the elements of the network, have been conceptually framed to help describing and understanding the Code as a network.

**Results:**

The network of the Code comprises 1,379 Nodes, which are connected with 11,276 Edges. The structure of the Code can be accurately described as a network, a mathematical structure that is better suited than any kind of linear text publication to reflect its structure.

**Discussion:**

Thinking of the Code as a network allows a better, in-depth understanding of the Code itself, as the user can navigate in a more efficient way, as well as to depict and analyze all the implied connections between the various parts of the Code that are not visible immediately. The network of the Code is an open access tool that could also help teaching, using and disseminating the Code. More importantly, this network is a powerful tool that allows identifying *a priori* the parts of the Code that could be potentially affected by upcoming amendment and revisions. This kind of analysis is not limited to nomenclature, as it could be applied to other fields that use complex textbooks with long editing history, such as Law, Medicine and Linguistics.

## Introduction

The application of standard, international rules of nomenclature (henceforth: the Rules) allowing unambiguous and universal communication about taxa is of paramount importance for the study, preservation and management of the entire biodiversity of our planet. Since the times of C. Linnaeus, scientists have attempted to describe life by using Latin and latinized words to name taxa and communicate their findings in a formalized way. Those names are used every day by scientists, teachers, students, in professional and personal life, and new names are created at the same time. To put it into perspective, Costello, May & Stork (2013) estimated that 5 ± 3 million species are present on Earth, 1.5 million are already described and named, and 17,500 of them are being described and named every year. An important part of this colossal taxonomic effort hinges upon universal Rules of nomenclature. These international Rules are grouped together forming separate Codes for different groups of species, i.e., animals, plants, fungi, etc. In this paper I provide a case study on the zoological Code, but the same method (with arguably similar results) can be directly applied to the other Codes as well. As these Codes mostly come from the same Linnean origin and largely have similar structure and logic (but some clear differences as well), I am confident that the results of this study are relevant to all fields of biology that deal with nomenclature.

The creation and application of taxon names for animals is based on a set of Rules that has been formalized as the International Code of Zoological Nomenclature (henceforth: the Code), led by the International Commission on Zoological Nomenclature (henceforth: the Commission). The current version of the Code (ICZN, 1999) was formed through a long history of discussions, revisions and amendments (Melville, 1996; Preface to the 1999 edition of the Code). The Code is mainly formed by a series of 90 Articles (with their Sub-Articles, Examples and Recommendations), together with the Preamble, the Glossary and three Appendices (ICZN, 1999: explanatory note to the Code). Zoologists and paleontologists from all over the world use the same stringent set of Rules as objective standards for the proper formation and application of nomenclature. However, the application of the rules of the Code is not always an easy task, as the language of the Code is relatively heavy and complex for non-specialists. Also, the structure of the Code makes sometimes its application difficult, as it is not an hierarchical one and it has been kept unmodified during the various revisions of the Code (Dubois, 2011).

Further complications might be imposed by additional actions of the Commission, which can use its ‘plenary power’ in various ways. First, the Commission is entitled to publish Declarations on specific parts of the Code—when these Declarations are ratified, they constitute a formal emendation of the Code (Art. 80.1, ICZN, 1999). Also, the Commission is entitled to publish Opinions on specific works, names or acts (Art. 80.2, ICZN, 1999)—these rulings apply only to those specific cases and do not form part of the Code (Art. 80.5, ICZN, 1999). As such, Opinions are not added in this network for the moment, but it might be a useful addition for the future, aiming at an even more inclusive nomenclatural network. Finally, the Commission is entitled to publish Official Corrections as Opinions (Art. 80.3, ICZN, 1999).

All these changes further complicate the structure of the Code, at least as a continuous flow of text. Driven by this issue, I set out to analyze the structure of the Code by using a network analysis. A network analysis is a powerful tool to reveal relationships between entities and as such its application has reached a wider spectrum to even include text data. For example, a network analysis has been used to connect trade agreements between countries (Alschner, Seiermann & Skougarevskiy, 2017), Supreme Court decisions (see http://law.ubalt.edu/faculty/scotus-mapping/), or the connections between the legislations of countries in the European Union (Koniaris, Anagnostopoulos & Vassiliou, 2017). A main objective of this paper is to use the underlying network analysis of the Code, in order to present a tool that can be used as a companion to the Code, for navigation and further analysis. The conceptual framework behind this network analysis is also explained in detail in the Supplemental Information, because it is important for the proper understanding of the methodology implied herein. Finally, a secondary objective is the discussion of some of the immediate observations on the structure of the Code, as they emerge from the network analysis.

I find that the structure of the Code is not reflected properly in its current presentation as a continuous text and the use of the underlying connections of the network is useful for the proper understanding and application of the Code. In fact, the Commission has described the Code as a “[…] *network of interdependent Articles*” (ICZN, 1999: Introduction), so I hope that this effort will be able to properly depict this underlying idea. I also highlight how the full understanding and use of the Glossary of the Code is essential for the proper comprehension and application of the rules.

The version 1.0 of the network is made available online in open access (OSF repository, http://osf.io/rjq7n). As such, this network of the Code can be used for the every-day application and practice of nomenclature, teaching and education of its principles to students and to solve complex nomenclatural cases. As the revision of the Code is a primary concern of the Commission (ICZN, 1999: Introduction), this network might help significantly in future revisions of the Code, as it can analyze rapidly and efficiently the potential effect of adding/removing/editing articles and other parts of the Code. In a broader perspective, this modern approach will make nomenclature and taxonomy more inviting and able to reach wider audiences within and outside the scientific community.

Perhaps the existence of this network will not make any difference for the experienced users of the Code—they already familiar with its structure and know how to use this text. But I sincerely hope that it could do wonders for students, starters, publishers and even opponents of the Code, to try to better understand and use it.

The name of this tool resembles a binomial. The basic tool and idea is called Neticon (after the initials of the phrase Networks of the International Codes of Nomenclature). The first example, presented herein, deals with the International Code of Zoological Nomenclature, and is therefore named as Neticon zoologicon. Different versions of the other Codes of Nomenclature will be published in the future, under their own kind of ‘binomial’.

### Methods

The methodology of a network analysis can be used for systems outside the strict sense of a network, after successful conceptual transformation of the system to resemble a network. For example, Esteve Altava (2013) used network techniques to analyze and describe the vertebrate skeleton. Therefore, the various conceptual decisions in applying the methodology of a network analysis to analyze the text of the Code are explained in detail in the Supplemental Information, together with explanations on the application and use of the various network metrics and descriptors used herein. The network was created with the free software Gephi 0.9.2 (Bastian, Heymann & Jacomy, 2009). Information on the contents of the Code was retrieved from the online version of the Code (https://www.iczn.org/the-code/the-international-code-of-zoological-nomenclature/the-code-online/), including the 2012 Amendment and Declarations 44 and 45. The 2012 Amendment (ICZN, 2012) presented the changes in various articles in order to allow publication of names and nomenclatural acts in electronic-only journals. Declaration 44 (ICZN, 2003) amended Art. 74.7.3, whereas Declaration 45 (ICZN, 2017) added some Recommendations to Art. 73 and a Glossary term. As these three documents modified the current version of the Code, they needed to be included in the final version of the network.

The main paper deals with the main conclusions that result from this analysis, without going into detail regarding the methodology of the network analysis. This detailed information is given in the Supplemental Information. I need to stress that the information provided as a supplement is fundamental for anyone looking to get a deeper understanding of the method. Note that I deliberately moved most of the specific information and terminology related to the network analysis as a supplement, to avoid adding unnecessary layers of complexity to this work.

## Results

**Figure 1:**
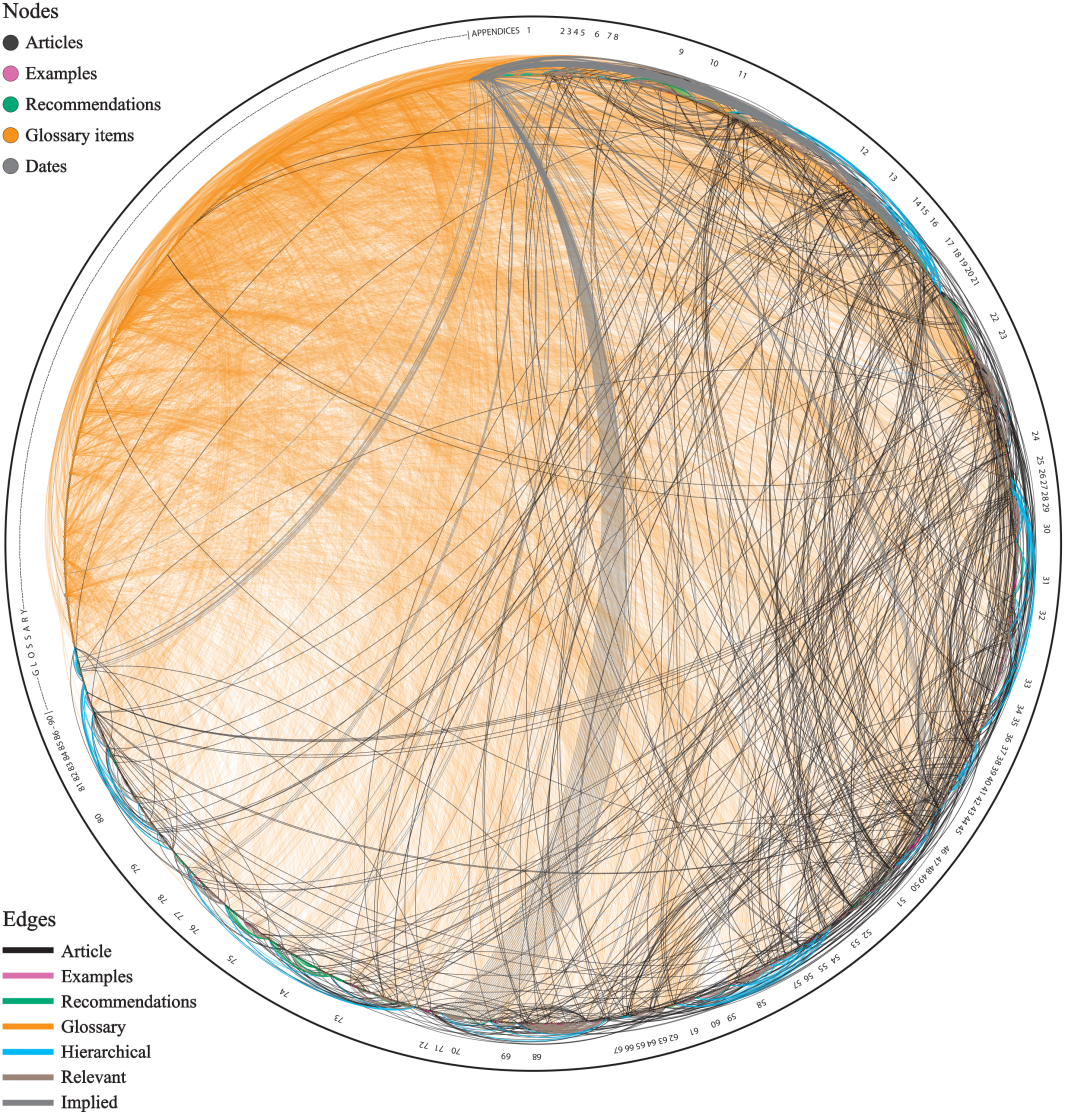
The ‘eye’ of the Code. Circular network diagram depicting the connections between the various elements of the Code. Articles are ordered clockwise according to their position in the current structure of the Code. The diagram indicates that the Code is not a continuous-flow text and has a complicated structure. Also the diagram indicates the importance of the Glossary (orange), which connects with the entire Code. Numbers of the outer circle indicate the main Articles of the Code.

**Figure 2:**
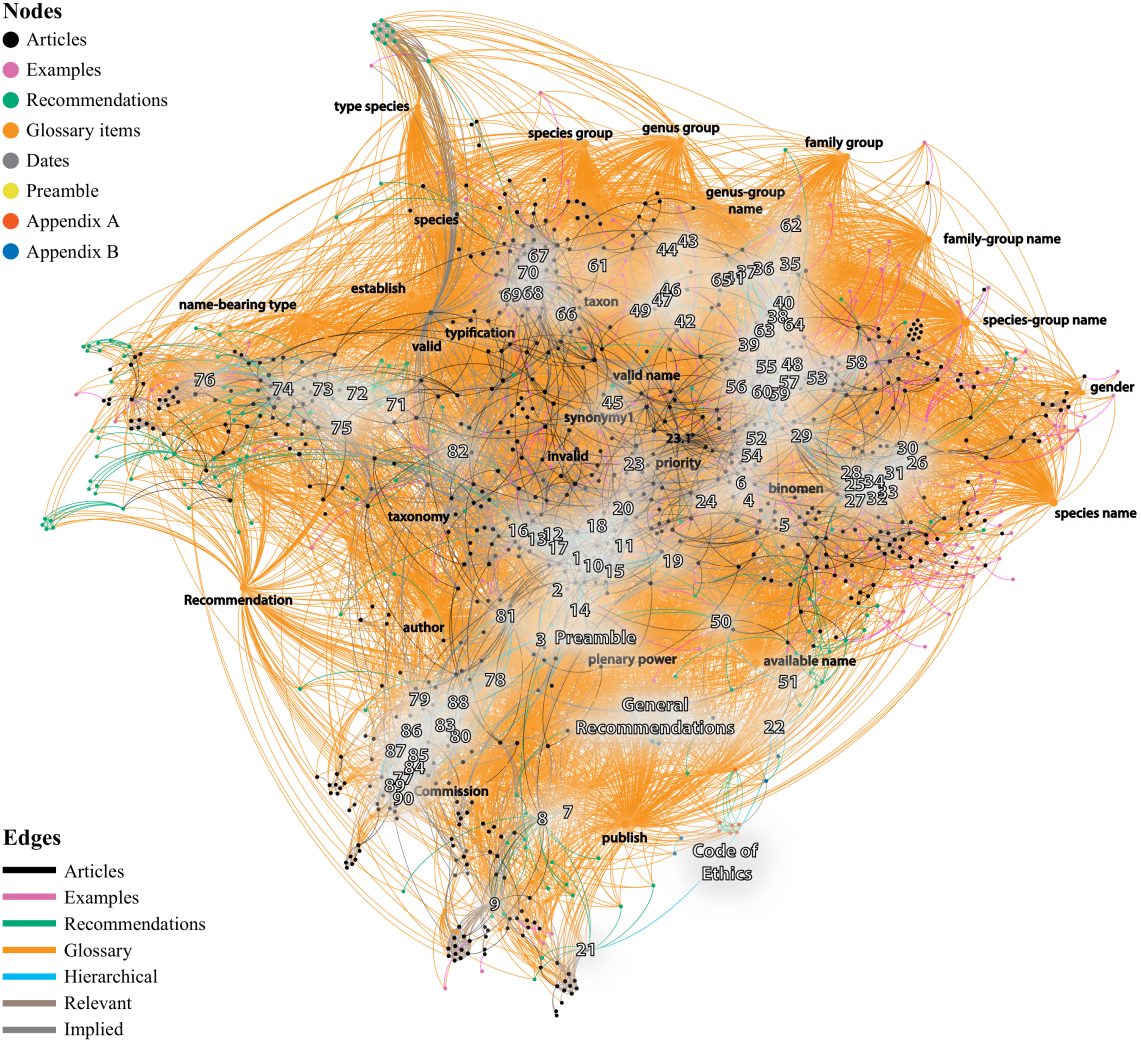
The ‘universe’ of the Code. Network depicting the structure of the Code under the Force Atlas 2 algorithm (see text for details). Articles and important Glossary terms are indicated.

The detailed results of the network analysis are given and discussed in the Supplemental Information (and Figs. 1 and 2 therein). Based on the successful conceptual application of the methods of network analysis to the continuous body of text of the Code, the network of the Code has been constructed. More than 51,000 words, grouped into 90 Articles (containing 754 sub-Articles, 129 Recommendations, and 129 Examples), 333 Glossary terms, and accompanied texts form the first version of the Neticon zoologicon which consists of 1,379 Nodes and 11,276 Edges. In-depth analysis of the network and the metrics that can be calculated from it, allows a better understanding of the structure of the Code. Before I discuss the major applications of this new tool, I would like to devote some space to describe the Code as it emerges from the network analysis. Because of the extension and complexity of the text of the Code, I think that it is difficult to grasp and conceptualize the Code as one “body”. The Neticon helps attributing some characters to the Code, much like describing other abstract concepts such as a social network.

For a body of text that is often attacked for its complexity, an interesting result of the analysis is that the Code as a network is actually quite simple. There are significantly fewer connections than possible, with only some clusters or hubs of Nodes, which increase the overall flow of the network. The Code, much like the social network that the reader is part of, is a scale-free network, where some parts of it tend to form more closely connected hubs. These deal with the most important concepts of the Code, like taxon, author, available name, species-group names, publish, species-group, species, species name, Commission, and the Principle of Priority. Although the Code is much more, the analysis of the network reveals that a significant part of the text of the Code is devoted to the process that allows authors to publish available names of taxa which then compete for validity via priority. The most important Article of the Code is Art. 23.1 (Statement of the Principle of Priority), having the most connections and occupying the most central position in the network.

The simplicity and good connectedness of the network of the Code reveals—in striking difference with the popular belief—that the interaction between the zoologist and the Code is actually quite efficient. I think that this is the most important result of this analysis. With the Neticon as a guide, any user of the Code would need to check only four parts of the text of the Code, on average, to reach a conclusion! The maximum amount of parts of the Code that a user needs to check is only eight! In other words, to answer any nomenclatural question the user should check, as an example, at most two or three Articles, with one or two Examples/Recommendations and two or three Glossary terms. This is not an empirical conclusion. This is the result of the mathematical network analysis of millions of connections between the various parts of the Code. Regardless what the reader might think—based on previous interactions with Code or even by reputation—the network analysis has been through all possible ways to “read” the Code and found out that if you start on any part of the Code you will have to read at a maximum seven more parts of the Code or only three on average to reach a conclusion on a specific question. If it takes more steps, it is because the user reads the Code without using the underlying connections of the network as a guide. Of course this only refers to logical connections that are already implemented in the Code; it is quite likely that the user might look for an answer to a specific question that is not covered in the current text of the Code. Therefore, the Neticon could be used to re-evaluate the majority of nomenclatural published works, Cases, and Opinions and identify new connections that might have to be made (and which exactly these connections should be).

Therefore, my main argument coming out of this analysis is that networks are extremely efficient ways to analyze and use heavy and complex legislative texts like the Code. In the following Section I discuss further this result with some more particular examples.

## Discussion

### The structure of the Code as a continuous body of text

As it has been mentioned already, the structure of the Code has been criticized several times, and extensive re-structuring is deemed necessary (see Dubois, 2011 for example, and references therein). However, the network analysis herein allows identifying the real problem with the structure of the Code. This is revealed when all articles are placed in their order of appearance in the text, with their connections made visible, as in the circular diagram of Fig. 1. Whereas I admit that this figure could be mistaken by someone as a ball of yarn, it shows something really important: the underlying connections between the Articles of the Code (dark lines) and the main nomenclatural concepts (i.e., the terms of the Glossary; orange lines) completely override the flow of the continuous body of text. And as these lines actually represent the interaction of the user with the Code—whether this refers to page-turning of a book or scrolling on a screen—it means that the current presentation of the Code as a continuous body of text does not serve the user. My point here is that, no matter what, it is impossible to present *this* Code as a continuous body of text following the current numbered order of the Articles. This is because the Code does not have the structure of a ‘normal’ book (i.e., with beginning, middle, and an end), but it actually has the structure of a small-world network. It is like if someone wants to place in a single line all the members of his/her social network. This is impossible, as relationships will start to diverge, forming closed or open loops and hubs. The same happens with such cross-referenced texts like the Code.

**Figure 3:**
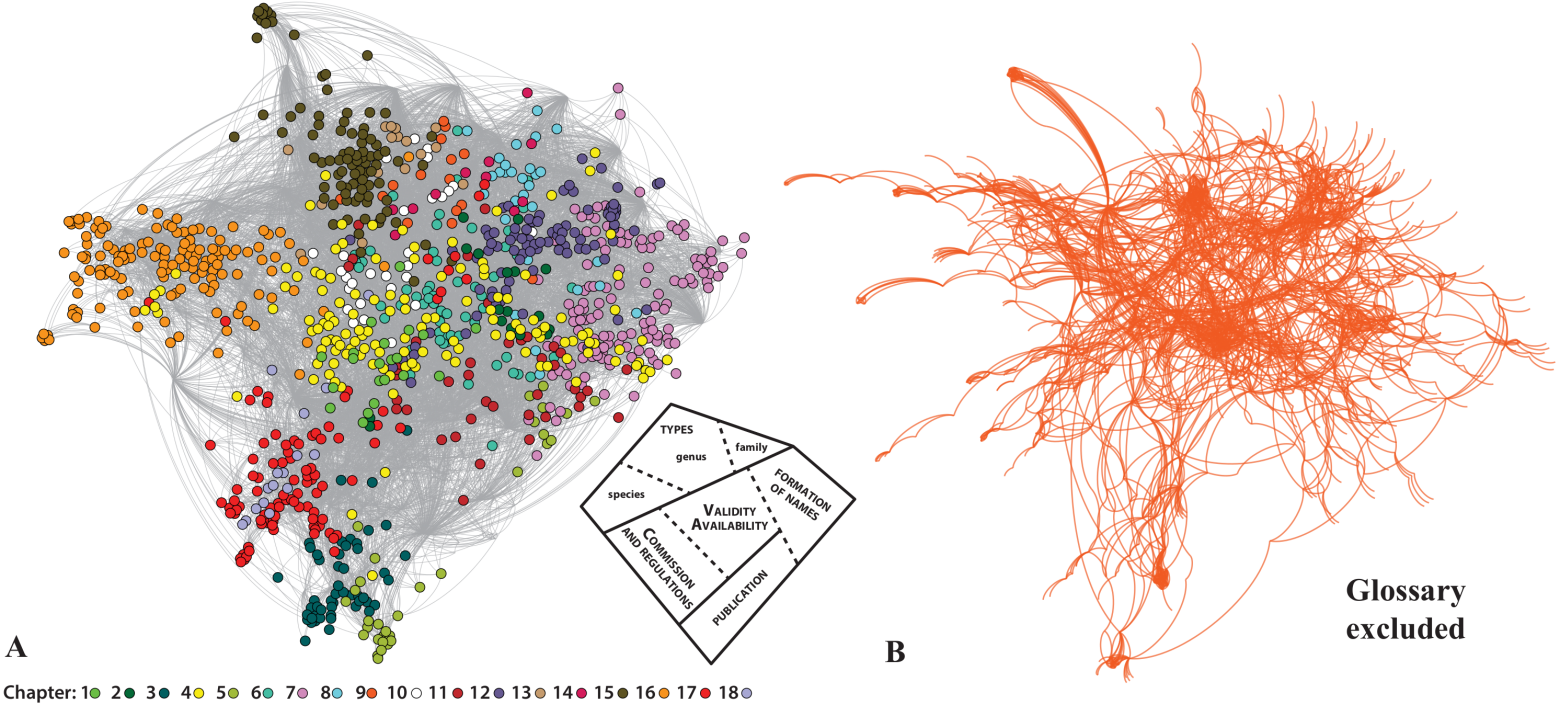
The analysis of the internal structure of the Neticon in a Force Atlas 2 layout. (A) The structure of the network with the various. Articles colored according to the Chapter they belong in. On its bottom right corner, a simplified diagram shows the thematic areas of the network. (B) The same network, if the Glossary is removed.

The actual structure of the Code—once the focus is on the connections between the various parts of the Code—is better depicted in Fig. 2, by using a layout algorithm that brings together the Nodes of the network based on their connections (Force Atlas 2 algorithm; Jacomy et al., 2014). Starting from the spatial center of the network where Art. 23 holds a central position, we immediately see that the remaining Articles forming several hubs around it. In some cases, these hubs are also expressed in the current presentation of the Code as a book: for example, Arts. 7–9 on publication criteria are indeed close to each other; but this is rarely the case. Perhaps the most clear example has to do with Arts. 36, 43, 46 (dealing with the Principle of Typification on family, genus, and species-group names), which although form part of different chapters of the Code are actually much closer together thematically. Seen at the Article level (Fig. 3A), this argument is much more obvious. For example, the Articles of Chapter 4 (Arts. 10–20; yellow color in Fig. 3A) which deal the criteria of availability, are widespread covering the entire ‘width’ of the network. This result is easy to understand, as the existence of available names in nomenclature is fundamental. Another example deals with the Articles of Chapter 10 (Art. 45–49, on species-group taxa and their names; white color in Fig. 3A), which are placed in between Chapter 16 (types in the species group), Chapter 15 (types in the genus group) and the Chapter 7 (on the formation and treatment of names). This is because the species is fundamental for the definition of types in the genus group (i.e., type species) and the genus name affects the formation of the species name. For example, Article 48 is much closer to the Articles of the Chapter 7, than to the Articles of its own Chapter. As Article 48 is about the change of generic assignment and includes concepts like gender ending and combination of species-group names it is quite relevant with the concepts of Chapter 7.

Another part of the Code that deserves some special mention is the Glossary. Article 89 dictates that our interpretation of the Code should be based on the meanings of words and expressions as explained in the Glossary. In the Preamble of the Code it is also stated that “[…] *this Preamble and the Glossary are integral parts of the Code’s provisions*”. These references alone should be enough to express the importance of the Glossary; but I am not sure if this is evident to many users. For example, there are terms that are almost only mentioned in the Glossary. The term ‘*nomen nudum*’ is only mentioned in the main text of the Code in the Example of Art. 51.2 and nowhere else in the Code! But nevertheless, the network analysis reveals that this term has a notable direct and indirect presence in the entire Code (it is an unavailable name). The connections of the Glossary extend to the entirety of the Code (see orange lines in Figs. 1 and 2). Without the Glossary, the overall structure of the Code would be largely the same (see Fig. 3B) because the Glossary has no legislative weight on the Code. However, the network analysis tells us that a Code without the Glossary would be much difficult to navigate and would require much more effort to reach a conclusion (see Supplemental Information).

Summarizing, the structure of the Code, when we focus on the connections between the Articles (Fig. 3A inset), is significantly different than the one proposed in the book. The most central part of the Code deals with availability and validity, and there are also well-defined areas regarding Types, Formation of Names, Publication criteria, and Commission-related information. Whereas some of them are accurately reflected in the book structure of the Code (e.g., Commission-related Articles), others are mixed (e.g., Articles of the Chapter 10 on the formation of names in species-group taxa are much closer to the Articles of the Chapter 16 on the types in the species group). These main areas of the network, namely the publication process and the subsequent availability of names, name allocation via typification, and name validity, represent important concepts that in many cases override the current presentation of the Code as a book. Dubois (2011) and references therein calls them ‘storeys’ or ‘floors’ of the ‘nomenclatural house’, criticizing correctly that the current structure of the Code does not accurately highlights this fundamental process. The results of the Neticon strongly concur with Dubois’s views, as the nomenclatural process is recovered in the Neticon (see Fig. S3 for a detailed analysis). The network analysis also allows observing the interconnections and overlap between these four stages. Still, the first part of the process, namely the publication and availability of names, overshadows the rest in the text of the Code. Clearly, without the first floor we would have no building at all.

I hope that I have provided several examples where the current structure of the Code depicts well the logical connections and cases where the current structure fails to do so. In principle, I would say that the network analysis herein provides good support on the criticism of Dubois (2011) and references therein on the structure of the Code. I am confident that the Neticon could help identify all these problems and assist in future changes in the structure of the Code. But I cannot say with certainty how difficult this task might be or even if it is feasible to do that. This is because I am convinced that the Code cannot be accurately presented as a continuous text. Perhaps it is not necessary to radically change the structure of the text of the Code. This text is a reference to past versions as well and should be seen as an historical and standard reference. But with the companion of the Neticon, it would be easier to navigate through it. Also, the Neticon could help identifying subsets of relevant articles and thus create smaller, autonomous, case-specific sub-Codes in seconds, with their appropriate structure. Just to give an example, with the Neticon we could create in a semi-automatic way subsets for a topic like the formation of names based on Ancient Greek, and with some extra editing work to present it as a simple continuous text (Supplemental Information 3). In other words, in my opinion the Code needs to remain a legal document, accompanied by simpler texts and informative manual that can be read as a continuous text; the Neticon can help completing this task.

### Navigating and using the Code

Certainly, a major use of the Neticon is the ability to navigate in a more efficient way. The analysis shows that if we trust and follow the underlying connections of the text of the Code as network, our interaction with it is efficient and minimal. The network itself could be directly used for navigation, via the web version or the original Gephi version (see Methods and Supplemental Information). Another possibility is to further enhance the online version of the Code with the information from the network herein. I think that this kind of interaction with the Code via a network on a computer screen is fundamental to “break the ice” between a beginner and the Code, which at a first glance looks like an impossible mountain to climb. The user is simply invited to interact with Code, in a way that was not available before. Accompanied by simple exercises to teach nomenclature with the use of the network might help to dissuade the notion that nomenclature is only accessible by some specialists.

### Understanding the code

For those interested in a more theoretical way, the network could increase their understanding of the Code and reveal underlying connections that might not be immediately evident. A more experienced user could use all the descriptors and metrics of a standard network mathematical analysis, which provide a quantitative way to measure and compare qualitative concepts like nomenclature (see Supplemental Information). Understanding each article of the Code individually, as well as its placement and position within its immediate surroundings and the Code as a whole, can help to gain a deeper understanding of the Code.

Also, and as the Code is an ever-evolving text for decades, an historical analysis of the “evolution” of the Code through its past versions, amendments, declarations and changes could provide valuable information on how and why the Code is what it is today. This historical analysis, via networks, could also help identifying the key changes that resulted in the current structure of the Code: how each version changed the structure and the system descriptors? Did the Code became more complex or less complex after each version? How each version affected the importance of several important Articles?

Another potential analysis would be to connect the Code with all published Opinions. Although the Opinions apply to specific cases and do not form part of the Code (Art. 80.5, (ICZN, 1999), it might be interesting to see how each Opinion and all as a whole, ‘attaches’ to the Code itself, via the mentioned Articles and terms. This kind of analysis could help identifying areas of the Code that constantly attract the necessity of the ruling of the Commission and discuss any potential changes to avoid that. As Opinions are issued in cases where the Code cannot be applied objectively or universally, reducing the necessity of actions by the Commission would improve the application of the Code.

### Revising the code

**Figure 4:**
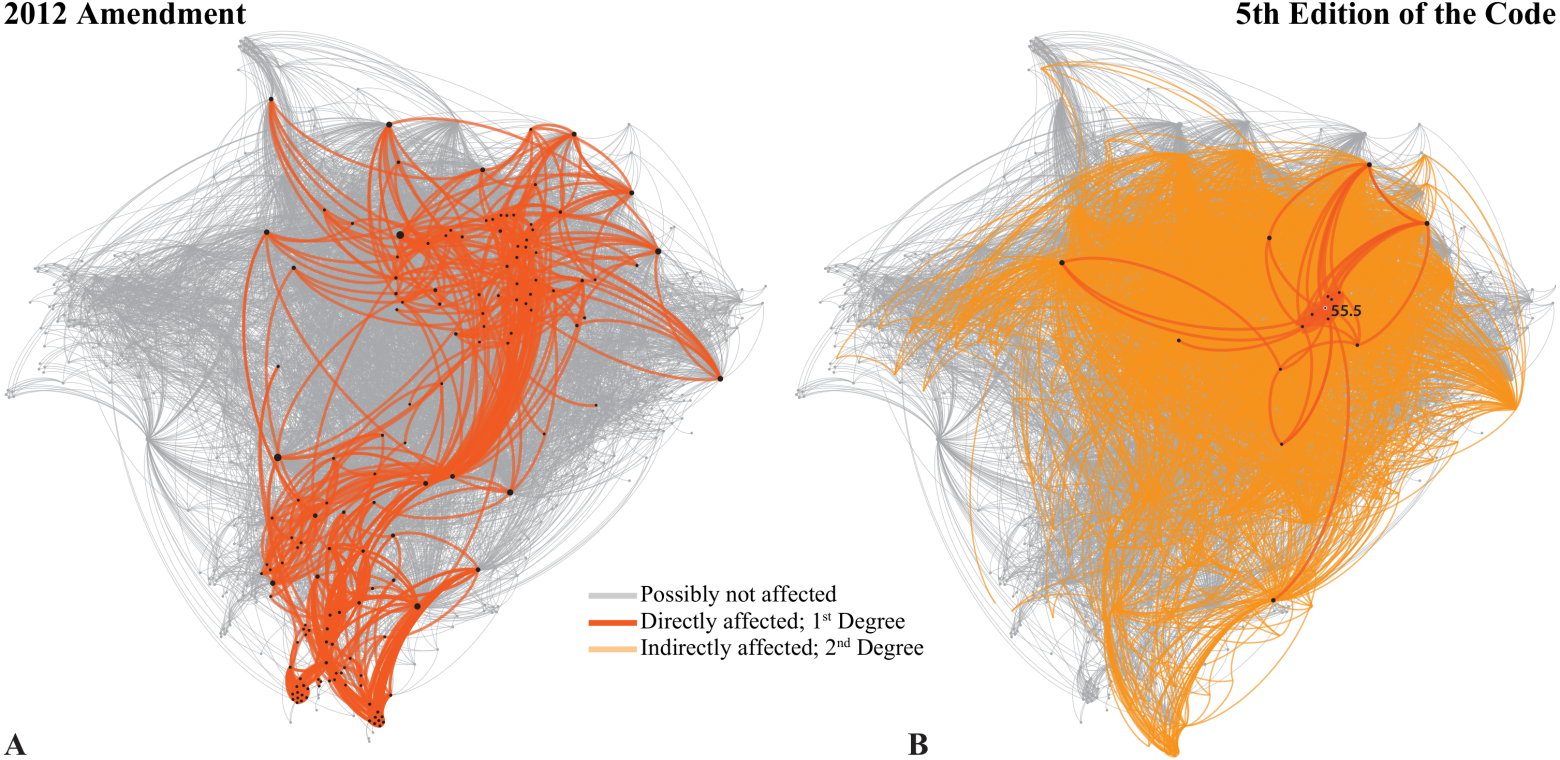
The Neticon can be used to help revising the Code. (A) The potential effect of the 2012 Amendment on the Code. (B) The effect of deleting Art. 55.5, as preliminary proposed by the Editorial Committee of the future ‘fifth edition’ of the Code. See text for further details.

Perhaps the most significant contribution of this tool is that it could assist significantly in revisions of the Code. In 2012, the Commission introduced a series of amendments in Articles 8, 9, 10, 21 and 78, in order to allow publication in electronic form. Several critiques on this Amendment have been published (Dubois et al., 2013) and the Commission has briefly replied to some of them (ICZN, 2013). If we try to analyze the potential effect of those changes in the Code, we find that immediately one third of the Code could be directly affected (Fig. 4A; using First Degree connections). If we extend this to the Second Degree, then the amended parts of the Code could influence more than 90% of the Code. This is because these amendments deal with concepts that, through the Glossary, extend their influence on the entire Code. Therefore, the changes introduced by the 2012 Amendment could potentially affect the entire Code. Of course, this does not mean that all these ‘potentially affected’ areas need to be revised or will indeed be affected. But the Neticon can at least signal those areas so that they can be thoroughly checked during revisions (see Supplemental Information, Ego network filter).

We are now going towards the ‘firth edition’ of the Code. An Editorial Committee has been selected, bound to work on several issues as explained on the preliminary information given at http://iczn.org/content/editorial-committee. I firmly believe that the use of the network could prove to be an essential tool for this task and any other revision, as those proposed by the Linz Zoocode Committee (Dubois, Aescht & Dickinson 2016; Dubois & Aescht, 2016). The network could be used, first, to identify all the potentially ‘affected’ areas of the Code, based on the First, Second and Third Degree connections of the edited articles. Then, and when the final version of the new Code is ready, comparisons and tests can be made with the previous versions. With the assistance of the Neticon, it might be possible to provide better revisions and perhaps even avoid conflicts or mistakes due to underlying connections between the articles that are not visible immediately.

To illustrate this claim, I will perform an experiment with the forthcoming ‘fifth edition’. From all the proposed changes I choose to focus on one that is clear, which is the “[*p* ]*roposed of correction of Art. 24.1 with deletion of Art. 55.5*”. It is therefore clear that the Editorial Committee of the ‘fifth edition’ intends to delete Art. 55.5 and make the necessary corrections in Art. 24.1. Article 55.5 is directly connected (Fig. 4B) with 16 Nodes of the Code (1.18%) through 51 Edges (0.46%). This might seem as an extremely small effect, but some of these 16 Nodes are among the most important in the Neticon. Thus, if we see the Second Degree connections of Art. 55.5 through its so-called ‘Ego Network’ filter (see Supplemental Information for instructions), its influence could potentially reach one-third of the Code.

Before Art. 55.5 is deleted, all its possible connections (I would say at least those within its Second Degree Ego Network; Fig. 4B) should be thoroughly checked. This of course does not mean that all these Articles are affected, but some might be. For example, deleting Art. 55.5 would ‘cancel’ the higher-rank-precedence of family-group homonyms, which might also affect synonyms as well (Art. 35.5 on the “Precedence for names in use at higher rank”; note the almost identical title with 55.5) or even possibly the Principle of Typification in family-group names (Art. 61.2.1). These possible connections are revealed with the Ego Network filter (see Supplemental Information).

## Conclusions

In this work I present a novel approach for studying and analyzing complex texts—complex in the sense of the amount of cross-references between the parts of the text—through the use of a network analysis, as exemplified by the International Code of Zoological Nomenclature. This text, composed of a set of Rules and Recommendations for the application and stability of the scientific names of animals, is seen by many zoologists as a difficult and incomprehensible text. The current structure of the Code has also been targeted as non-logical (Dubois, 2011). My analysis reveals that, indeed, the current presentation of the Code as a continuous body of text does not reflect accurately the structure of the Code. The Code should be rather seen as a network of Nodes connected with a multitude of Edges, a concept that actually appears in the Introduction of the Code. The complex text of the Code can be broken down into approximately 1,300 elements connected through some 11,000 connections—these form a rather simple network of interconnected Nodes. When looking for an answer to a specific question, on average, the user should go through four parts of the Code; however, no more than eight parts need to be consulted to answer any question. It is remarkable that when the Neticon is used as a guide, results in a quite efficient and simple interaction with the Code.

This complexity and interconnectivity, of course, poses problems for zoologists not only in the every-day use of the Code, but also when it comes to the revision of any part of it. Because of the connections between the various parts of the Code, even the smallest change could potentially influence, if not checked in advance, a large part of the Code or remote Articles that do not seem connected at first. An experienced user of the Code could certainly see the immediate (First Degree) connections of any Article and some of the Second Degree ones. But it is extremely difficult to see beyond that with any traditional method. In several cases, the negative effect of these implied connections could remain unnoticed during the Amendment of the Code, only to be discovered later through the application of the amended Rules. The Neticon zoologicon is a tool that could efficiently help minimizing these negative effects. Therefore, a main advantage of this network is that it allows, for the first time, a control over the negative effects of upcoming revisions of the Code.

Although some specifically designed courses on Zoological Nomenclature exist (e.g., see for example courses on DEST, the Distributed European School of Taxonomy) the majority of zoologists perhaps have never received proper training on nomenclature. So, and besides this important contribution, this network could have an enormous contribution in teaching and learning the Code and its every-day use by zoologists. For example, the network could be used to create exercises and lessons to teach the use and application of the Code more effectively. These lessons should aim to provide a basic and solid understanding of the basic principles of the Code and its structure, before the user is ready to confront the legal text of the Code.

Also, this analysis could provide some insights on the current presentation of the Code as a set of continuous Rules and Articles. Although I prove that the current presentation of the Code does not reflect its connected structure, this does not mean that I suggest that the book of the Code has to change. I argue that we should not try to seek a way to perfectly present the Code as a book because, no matter what, it is impossible to do so. The Code is a network, not a continuous text. The full text of the Code should remain as it is, but it should be seen as a reference to search an Article, not as the source to learn and apply the Code. Instead, the network could be the primary source for the use and application of the Code, with the actual text as a companion. Also, the network could be used to automatically produce some, smaller, subsets of the Code to cover some specific cases. For example, as the Code has different sets of Rules for names published during different periods of time (e.g., for names published before 1930 and after 1931), the network could be used to produce the full set of Rules that are relevant for each case. So, the user could use the appropriate subset for each case. Similarly, other subsets could be produced to cover specific topics, such as the gender agreement, homonymy, priority or names published in electronic-only journals; the possibilities are endless, but this network could do all this hard work in seconds (see Supplemental Information). These subsets will be, of course, overlapping with each other and they will be based on the full version of the Code. But in this case, it will be faster and more efficient for the user to reach a conclusion via the appropriate subset, without having to go through the entire Code. These smaller subsets would also provide some sort of confidence to the regular user (by regular I mean users like myself that are not aware of every detail of the Code) that he/she has reviewed at least the most relevant parts of the Code before reaching a conclusion.

Seen as a network, the Code is no more a heavy, immovable text. Instead, the Code becomes a ‘living, evolving body’ of text, which could be vulnerable to even some minimal modifications, but at the same time resistant to large-scale changes. It is not a solid block of text that has been put there by some higher authority to govern the names of our beloved living and extinct species. It is a complex structure that reached its current form through decades of application, modification and criticism (Melville, 1996); those various stages in the ‘evolution’ of the Code are highly evident in its structure and in a way contribute in the inherent complexity of the Code. It is a complex structure, a product of the collective efforts of zoologists during the last centuries, which invites us to explore its morphology, understand its strengths and weaknesses and solve some of its problems. In such complex systems or networks, any attempt on changing their structure, without first fully understanding it, could have catastrophic consequences (see also in this respect: Dubois, 2010).

This is only a first step towards a full understanding of the Code, which hopefully opens the door to in-depth analyses with a final objective to produce a set of Rules of nomenclature that we can all be happy about. It would make sense to start investigating, with a network analysis, the ‘evolution’ of the Code itself, through its various stages, versions and amendments. Such analyses would highlight some of the main problems, as well as when and why they appeared. Continuing with this analogy with a living organism, I find it entirely understandable how and why new ‘species’ of Codes appeared, stemming from the Code itself and most certainly they will continue to appear. Whether these new ‘species’ of Codes are hybrids, parasites or eventually a ‘derived’ version of the current Code remains to be seen. But in that case, I am confident that the methods employed herein could provide useful insights.

The results obtained in this study, in my opinion, extend beyond zoological nomenclature. To some extent, other fields of biology that use binomial nomenclature face the same or similar problems and there is no doubt that a network analysis could help identifying them to considerable detail. Perhaps what we need is this shift in understanding that the Codes are not heavy and impossible texts but rather open networks. To try to focus in the lines that connect the concepts that nomenclature deals with, rather than those that divide them. And maybe that way we will be able to bring back everything together and present a single, unified, Code of Nomenclature.

I am optimistic that this modern approach will make nomenclature and taxonomy more inviting and able to reach wider audiences within and outside the scientific community. Clearly, non-scientists, laymen, legislators, and decision makers do not need to work with binomial nomenclature and its Codes. Their exposure to nomenclature usually extends to the simple use of binomials. But being able to identify and solve the problems with our Codes of Nomenclature will send a positive message. Also, I have no doubt that this network approach could be useful to other fields that use such heavy and complicated textbooks like our Code, such as Law, Medicine and Linguistics. Still, the Neticon zoologicon cannot actually give you the right answer to a specific question; the user still has to read, comprehend and interpret the Code, but hopefully this tool will be a good companion.

##  Supplemental Information

10.7717/peerj.8127/supp-1 Supplemental Information 1 The original network in Gephi format This can be opened and edited with the open-source software Gephi: https://gephi.org/.

10.7717/peerj.8127/supp-2 Supplemental Information 2 Supplementary Information This supplementary information file contains detailed information on the methods used in this work, including a detailed analysis on the conceptual framework on how the Code has been successfully transformed into a network (**Supplementary File 1**). Then, the main results of the analysis are given, with emphasis in network properties and calculated metrics (**Supplementary File 2**). **Supplementary File 3** contains an example on how the Code could be re-organized to create subsets on some topics. Finally, basic instructions on how to use this tool are inserted in the end of the file (**Supplementary File 4**).

10.7717/peerj.8127/supp-3 Supplemental Information 3 The network tool for offline use This zipped file contains a folder with the live network for offline use. Please unzip the file, and locate the file called ”index.html”. Please open it with any browser exc. Google Chrome. You will get a webpage with the live network, for navigation purposes. This version of the network cannot be edited. Exported with the built-in plugin in Gephi, developed by the InteractiveVis project of the Oxford Internet Institute.

10.7717/peerj.8127/supp-4 Figure S1 Diagrams of some of the descriptors of the network of the Code (A) Curves showing the variation of Degree and Weighted Degree across the Code. Articles are ordered according to their position in the current structure of the Code and a distinction between the main Articles and the Glossary is shown. (B) Histogram of the clustering of the Nodes. If the clustering of a given Node is 1.0 it means that this Node is connected to every other Node in its neighborhood. (C) Linear correlation between the Weighted Degree and the Clustering Coefficient. (D) Linear correlation between the Weighted Degree and the Closeness Centrality. (E) Histogram depicting the distribution of Degree in the network.

10.7717/peerj.8127/supp-5 Figure S2 The analysis of the Neticon in Force Atlas 2 layout (A) the structure of the network if the Glossary is removed. (B) The same network, but the Articles are colored according to the Chapter they belong in. On its bottom left corner, a simplified diagram shows the thematic areas of the network. (C) The distribution of the Nodes according to their Clustering Coefficient. (D) The distribution of the Nodes according to their Closeness Centrality. (E) The distribution of the Nodes according to their Betweeness Centrality. In C–E, the magnitude of the corresponding metric for each Node is indicated by the size of the Node and its color (red: high, orange: middle, blue: low).

10.7717/peerj.8127/supp-6 Figure S3 The nomenclatural process depicted in the Neticon A, the main stages of the nomenclatural process are mapped with different colors in the network, by using a central term and its 1tˆ Degree connections (Ego networks in Gephi). Each subset is separated in its own Force Atlas 2 network. B, the nomenclatural process starts with the publication of a name (bluish green). C, publication creates available names (vermillon). D, these names are allocated to taxa with name-bearing types (reddish purple). E, then, names enter nomenclature to compete for validity (sky blue).

## Additional Information and Declarations

### Data Availability

The following information was supplied regarding data availability: All necessary data associated with this project are available in the Supplemental Files. Data is also available at OSF: Vlachos, Evangelos. 2019. “Introducing a New Tool to Navigate, Understand and Use the International Code of Zoological Nomenclature.” OSF. November 2. http://osf.io/rjq7n.
