# Supplementary material for: Introducing a new tool to navigate, understand and use International Codes of Nomenclature
Source: PeerJ. 2019 Nov 25;7:e8127. doi: 10.7717/peerj.8127 (PMC6882414; doi:10.7717/peerj.8127)
Supplement: Supplemental Information 2 — This supplementary information file contains detailed information on the methods used in this work, including a detailed analysis on the conceptual framework on how the Code has been successfully transformed into a network (Supplementary File 1). Then, the main results of the analysis are given, with emphasis in network properties and calculated metrics (Supplementary File 2). Supplementary File 3 contains an example on how the Code could be re-organized to create subsets on some topics. Finally, basic instructions on how to use this tool are inserted in the end of the file (Supplementary File 4). [file peerj-07-8127-s002.pdf]

Introducing a new tool to navigate, understand and use the International Code of Zoological Nomenclature

Evangelos Vlachos

CONICET - Museo Paleontológico Egidio Feruglio, Trelew, Chubut, Argentina

Corresponding Author:
Evangelos Vlachos
Av. Fontana 140, Trelew, Chubut, 9100, Argentina
Email address: evlacho@mef.org.ar

Supplementary Information

This supplementary information file contains detailed information on the methods used in this work, including a detailed analysis on the conceptual framework on how the Code has been successfully transformed into a network (**Supplementary File 1**). Then, the main results of the analysis are given, with emphasis in network properties and calculated metrics (**Supplementary File 2**). **Supplementary File 3** contains an example on how the Code could be re-organized to create subsets on some topics. Finally, basic instructions on how to use this tool are inserted in the end of the file (**Supplementary File 4**).

The version 1.0 of the network is made available online in open access (OSF repository, osf.io/rjq7n).

Also, please visit the webpage at < http://staff.mef.org.ar/en/researchers/evangelos-vlachos/iczn-network > for further information.

Supplementary File 1:

Methods

The network was created with the free software Gephi 0.9.2 (Bastian *et al.* 2009). Information on the contents of the Code was retrieved from the online version of the Code (<http://www.iczn.org/iczn/index.jsp>), including the 2012 Amendment and Declarations 44 and 45. The 2012 Amendment (ICZN 2012) presented the changes in various articles in order to allow publication of names and nomenclatural acts in electronic-only journals. Declaration 44 (ICZN 2003) amended Art. 74.7.3, whereas Declaration 45 (ICZN 2017)[1] added some Recommendations to Art. 73 and a Glossary term. As these three documents modified the current version of the Code, they needed to be included in the final version of the network.

First, all Nodes were created manually for the entire Code, including the Glossary. For each separate piece of the Code a different Node was created, under the following types: Regular (regular Articles and Sub-Articles); Example (for the Examples of the Code); Recommendation (for the Recommendations of the Code); Glossary (for the Glossary items of the Code[2]); Dates (Nodes referring to important years for the application of the Code—e.g., 1758, 1930). Nodes were connected with Edges manually. The different kinds of Edges used herein are: Direct Article (to connect Nodes that are explicitly cited within the Code; e.g., see Art. 23.1); Relevant (to connect Nodes that are related—e.g., an Article with each of its Sub-Articles and their Infra-Articles if applicable); Glossary (connections of the various terms of the Glossary entries with each other and with relevant articles); Example (connections of each Article with its relevant Examples); Recommendation (connections of each article with its relevant Recommendations); Hierarchical[3] (hierarchical, structural connections between Articles that are included in the same Chapter); Hidden (implied connections because the various important dates of the Code[4]). When the connection between Nodes is a directed one, this was depicted also in the kind of Edge

[1] The changes of the 2012 Amendment and the Declaration 44 form already part of the online version of the Code, which was mainly used for the creation of this network. Declaration 45, on the other hand, has not been implemented yet in the webpage at the time of the creation of this network.

[2] In the cases where a Glossary item has more than one meaning (e.g., 'availability'), separate Nodes were created for each meaning, using the numbering of the Glossary (e.g., availability1, availability2, availability3).

[3] It should be noted that this kind of connections is not the same as the 'Relevant' one. A 'hierarchical' Edge is made after following the hierarchical structure of the Code and connects only the Articles included in a given Chapter in the way that the Code is presented. 'Relevant' Edges connect the Articles and the Sub-Articles according to their context and their hierarchical presentation is not taken into account. The purpose of the 'hierarchical' Edges is to provide an initial 'structure' of the Neticon.

[4] In several parts of the Code, various important dates are mentioned which connect parts of the Code that are tied by the same date. For example, Art. 1.3.6, 9.1, 11, 13, 67.4.1 and the term '*nomen nudum*" are connected to each other via the date 1930. However, this connection is not immediately visible and is revealed only after the inclusion of the Nodes on important dates; hence the term 'hidden'.

(Directed vs. Undirected). A Directed Edge was added when the text uses the expression "see Art. 23.1", or when the connection is implied with a modal verb like 'must' (e.g., "must satisfy the provisions of Art. 12" or a similar expression)—in all other cases the Edges were Undirected[5]. The use of different types of direction in Edges would not alter the final structure of the NETICON, but it would affect some of its features/metrics like the Degree of connections between the Nodes and the overall Average Degree. Different weights were assigned for the various kinds of edges, implying their importance. Parts of the Code that are not mandatory (e.g., Examples and Recommendations) or are explanatory (e.g., Glossary) should receive less weight compared to the main legislative text (e.g., Articles). These weights were assigned subjectively as: Relevant (3.0); Article (5.0); Glossary (1.0); Example (1.0); Recommendation (1.0); Chapter (2.0); Hidden (1.0). The idea of applying different weights to Edges is because I want to provide numerical focus on specific kinds of connections that I consider more important than others in the Code. The different weights are expected to influence both the final layout under some weight-specific algorithms (e.g., the Force Atlas 2 algorithm used herein: Nodes connected with heavier Edges would be more attracted) and some network metrics.

For the layout of the Code I used the following two types of layout included in GEPHI with the following settings: Circular (counter clockwise) and Force Atlas 2 (tolerance = 0.2; scaling = 100.0; gravity = 1.0; prevent overlap ON; dissuade hubs ON; edge weight influence = 1.0). In this Circular layout, the Nodes are ordered according to their continuous order in the text and their connections are visible but they do not influence the layout. In the Force Atlas 2 layout, on the other hand, Nodes that are connected with more Edges are placed closer together, forming hubs of Nodes that are more closely related to each other. The Force Atlas 2 layout depends only on the Edges that connect the Nodes (Jacomy et al. 2014). This is a classic force-vector algorithm having a linear-linear model that calculates the attraction and repulsion proportional to distance between Nodes, with a Barnes Hut optimization (Jacomy et al. 2014). Therefore it is ideal to depict the structure of the Code as inferred by the connections between its various elements. The networks were exported as svg or pdf files from GEPHI and further edited for stylistic purposes in ADOBE ILLUSTRATOR. Those files were used to create the figures herein. The web version was exported with the plugin SIGMAEXPORTER 0.9.0. The various versions of the network and all

[5] This was a conceptual *a priori* decision I made in order to depict the direction flow within the Code. A Directed Edge dictates a kind of mandatory flow direction. For example, Art. 8.3 directs you to read Art. 8.7.1, but not the other way round. Art. 8.5 states that in order to be published *sensu* the Code, an electronic work must satisfy all provisions explained in 8.5.1–3. In other words, a Directed Edge indicates a connection that the reader cannot ignore. Alternatively, with an Undirected Edge, the reader could read the connected Article, but this is not necessary.

associated files are available at <http://staff.mef.org.ar/en/researchers/evangelos-vlachos/iczn-network>.

Conceptual framework

Before describing and discussing the network of the Code, it is necessary to explain some basic concepts of a network analysis that are essential for the reader. Also, it is important to explain how some of these concepts are transformed when applied to the Code, compared to their traditional meaning in standard network analyses.

Interpretation of the network model

In this section I would like to introduce the reader to the basic concepts of the network models that are mentioned herein. Also, I attempt to translate the various Descriptors with definitions that could make some sense in zoological nomenclature. This is commonly done in cases where network theory is used to describe a system that is outside the strict interest of the method. For example, Esteve Altava (2013) had to explain how the mathematical network model descriptors could be interpreted in his anatomical networks of tetrapod skulls—Esteve Altava's (2013) work is used as a model and inspiration here. Any network comprises two kinds of elements: a set of Nodes that is connected by a set of Edges. Based on these connections, a series of Descriptors can be calculated, both for the elements of the network and for the entire system. Although a network is primarily a mathematical structure, it can be used to model many kinds of structures (see Esteve Altava 2013 for basic information and application of network theory to anatomical networks).

The Code can be depicted by these two basic elements as any other type of network. All the Articles, Examples, Recommendations, etc. are conceptualized as Nodes of different types. On the other hand, the explicit (those stated in the text) and implicit (those implied by structure, hierarchy and context) connections between the various parts of the Code are conceptualized as the Edges connecting the Nodes. The Edges represent the decision and action of the user to 'move' from one part of the Code to another. This is many times dictated by the Code itself (e.g., when the Code writes: see Art. 23.1), or by the user after following a logical procedure (e.g., this Article is about neotypes, so the user must go to the Glossary to check what is the meaning of the term neotype).

A single or separate networks?

A critical decision that one needs to make when analyzing complex texts as networks—like the Code—is whether a single network should be created versus a number of separate networks. Although I acknowledge that the Code is composed by a set of different kinds of texts (e.g., the Preamble, the Articles, the Recommendations, the Glossary etc.), these sets are clearly connected to each other.

The most important distinction between these different kinds of texts concerns their juridical impact. Of course, Recommendations and Examples, as well as the Appendices of the Code, have no immediate juridical impact—however, they do have indirect impact on the mandatory provisions of the Code because they help our understanding of these mandatory provisions. Both in the Explanatory Note on the Code and the Introduction (Anonymous 1999), it is stated that Recommendations and Examples form part of the Code (although are not mandatory), something that is evident in their presentation as well (they are 'hierarchically' or 'structurally' placed within the various mandatory Articles; the Recommendations are also numbered after the Article in which they are included). Therefore, I think that it would be a mistake to exclude them from the network. Their inclusion, however, does not suggest that they have the same gravity or importance with the mandatory parts of the Code—this is modeled with the various weights of the connections between them (see Methods). In any case, the user is able to automatically exclude these parts with the use of network filters.

What is not included in the network of the Code

Some of the information that forms part of the contents of the Code has not been included in the network as separate Nodes. In particular, the introductory Explanatory note and the Preface to the 1999 Edition, were not included, as it proved impossible to divide these respective texts into separate Nodes, at least by using the same criteria as below for the rest of the text of the Code. Similarly, the Constitution of the Commission is excluded from this version, as it does not form part of the legislative text of the Code and does influence our understanding of the contents of the Code. Finally, both the Summary figure and the Index are excluded as well. Both entities are, in some way, reminiscent to the network herein. The Summary figure represents a network-like hierarchical structure. This figure cannot be included in the network because it describes a process that is followed from the publication of a work to the validity of names and nomenclatural acts. So it is not directly relevant for the network herein. It must be noted that similar figures have been presented by Dubois (2011, Appendix 3). Therein, the Summary figure

of the Code is expanded, including or the different technical nomenclatural terms of Dubois (2011, Appendix 1; and references therein). The Index is actually a series of connections between the elements of the Code, similar to the connections made herein, but the current network is far more complete.

What is included in the network of the Code

The network deals with the entire text of the Code and aims to depict its underlying connections, on the basis of the recommendations available to the user to navigate the Code. Therefore the entire text is divided in numerous parts, forming the Nodes of the network. In the majority of the cases the Code itself provides the proper separation of the text into Nodes: each independent part of the text of the Code, defined by a numbering or any kind of heading, forms a Node. In particular, the Nodes of the network contain: titles of Chapters; general titles of Articles; title and text of each Sub-Article; title and text of each Recommendation; title and text of each Example; title and text of each Glossary item. Thus, if the user exports all the Nodes in their entirety, he/she would get the complete text of the Code. Besides the main text and the Glossary, both Appendix A (Code of Ethics) and B (General Recommendations) have been added to the network. These two Appendices do not form part of the Code (Explanatory Note, Anonymous 1999), but I choose to include them in the network of the Code for several reasons. First of all, my intention is to provide the most inclusive, extensive representation of the Code itself, trying to include as much relevant information as possible. Also, both Appendices are presented in a way that allows a clear definition of Nodes. Finally, both Appendices contain concepts that are directly and indirectly connected with the rest of the Code and are indispensable for any zoologist. So, I believe that it would be a missed opportunity and a mistake not including them in this project.

One more kind of Node has been created, although not included in the Code as such: the various important dates for the application of Articles (e.g., 1757, 1758, 1930, etc.)[6]. This allows an even more detailed depiction of the structure of the Code. For example, names that were published before 1931 have relevant Articles spread out throughout the Code. A total of 54 elements of the Code, including Articles 1.2.1, 11, 11.4.3, 12, 12.1–3, 13.3.1, 23.3.2.3, 42.3.2,

[6] Other terms which could be useful to provide some additional groupings in the network (e.g., species, genus, family, etc.) are already included as terms in the Glossary. So the user can use the Glossary Nodes to create those subgroups. Adding Dates as attributes for the various Nodes would not work so well, because the majority of the Articles are applicable for names and publications published at any date. It would create, in my opinion, the false idea that only the Nodes labeled with a specific date are relevant for taxa or works published before/after that date. These Nodes are added in the navigation tool for convenience.

67.2.2, 68.2.1, 68.5, 69 and several of its Sub-Articles and Recommendations and 72.5.1 are implicitly or directly connected to each other because they deal explicitly with names published before 1931. Therefore, this network provides the possibility to navigate through the Code by using these various important dates. This kind of Node does not form part of the original text of the Code but nevertheless appears in several of the titles of the Chapters. Also, the Code explains how these parts are there because they apply to actions that were taken in the past[7]. So, I hope that the decision of adding these Nodes will not be seen as a stretch nor will it affect the homogeneity of the Nodes of the network.

Now that the Nodes and Edges are properly defined and conceptualized, a mathematical model network can be constructed and analyzed. Any network is interpreted via a series of Descriptors or indices. Those referring to the main elements of the network (Nodes and Edges) are called Element Descriptors. Those referring to the entire network as a whole are called System Descriptors.

Descriptors

Element Descriptors

This kind of Descriptors is used to describe the two elements of the network, namely the Nodes and the Edges. Only the most relevant ones will be mentioned. Also, it is important to mention that these Descriptors should always be analyzed together if one wants to evaluate the importance of a given part of the Code. These Descriptors can help answering questions like: '*is this Article/concept important?*', or '*do I need to read many more Articles to understand what this Article is about?*'.

Degree and Weighted Degree. The connectivity of each Node is simply calculated as the sum of all the Edges that connect this Node with other Nodes; this is the Degree (D). If the Edges have different weights (as it is done herein), the Weighted Degree (WD) is the sum of the weights of all the Edges that connect a given Node to the network. The D and WD are two basic Descriptors that partly describe the importance of a part of the Code—if a given part is connected with many other parts of the Code, it might be more important than other parts that are less connected[8].

---

[7] "[T]he principal cause is the requirement that rules that are mandatory for current acts and new names must not upset actions taken by past generations operating under different and less restrictive, nomenclatural rules or conventions" (Anonymous 1999: Introduction).

[8] The Article with the highest D and WD is Art. 23.1, i.e., the Statement of the Principle of Priority. Most certainly, this is one of the most important Rules of the Code.

However, whether this importance is of broader significance or not it is measured by other Descriptors mentioned below.

Clustering Coefficient. The Clustering Coefficient (CCo) of a Node measures how it tends to cluster together with its neighboring Nodes, by calculating the ratio between the connections of the Node with its immediate neighbors and the total number of possible connections between these Nodes. The CCo in the NETICON would show the conceptual relation of each element of the Code (e.g., an Article) and its immediate neighbors. If a part of the Code has high Cco (or even its maximum value, 1.0), it means that all the Articles connected to it are relevant to each other. In other words, the reader must read and understand all or most of its immediate connections—usually these are Articles with few connections as well (low WD correlates with high CCo, see Results). A part of the Code with high CCo is also a part with 'local' importance, as the relevance of this part rarely extends to its immediate neighbors[9]. On the other hand, parts of the Code with low CCo (and high Degree) are those that have broader significance and represent concepts that everyone should be familiar with[10].

Centrality. Centrality is a key concept in network theory and refers to the influence that a given Node has on the network and thus measures its importance in another, complemental, way. Two Centrality indices will be further used herein, the Closeness Centrality (ClCe) and the Betweenness Centrality (BCe). In several cases the reader might find mentions of another Centrality measure, the Degree Centrality: this is simply the Degree of a Node as explained above. Note that whereas the Degree only describes the amount of connections of a given Node, the other Centrality indices try to measure importance based on the position of this Node in the network as well. These are different and complementary methods of measuring the importance of a Node in a network.

The ClCe is calculated as the average length of the shortest path[11] between this Node and every other Node of the network. Thus, ClCe is a major measure of the importance of each Node

[9] The Sub-Articles of Art. 8.1 have high CCo and low D and WD, as they are tightly connected to each other but not to other parts of the Code. Indeed, these Sub-Articles represent indispensable criteria to be met so that a work is considered published under the Code. Also, most of the terms used in this Article do not need specific definitions in the Glossary. Thus, the reader, in order to comprehend what this Article is about, only needs to read this particular page of the Code.

[10] This is quite evident for concepts of the Glossary like author, taxon, species, available name, etc., which are relevant for the entire Code.

[11] The shortest path is the minimum steps that are needed to be made to reach from one Node to another, of course always following the connections of the network.

of the network. A part of the Code with high ClCe would mean that it represents a central concept or Rule of the Code[12].

The BCe measures a different kind of Centrality. It measures how many times the Node in question acts as a 'bridge' along the shortest path between two other Nodes. Of course it is common that important Nodes (with high D, WD, ClCe, etc.) will also have high BCe. However, this measure might reveal Nodes whose importance is not immediately revealed by other, more direct, Descriptors; this is what interests me for the NETICON. As such, parts of the Code with high BCe[13] would represent concepts or Rules that one needs to understand before applying the Code, because those concepts or Rules are more probable to come up during reading than others, while the user is navigating through the Code. In other words, a part with high BCe has great conceptual influence to its connected parts. This is a key Descriptor to measure importance.

System Descriptors

When the various Element Descriptors, like those mentioned above, are combined for the entire network, several general parameters can be calculated. Those metrics describe the whole network and as such are called System Descriptors. The most relevant are listed below.

Density. The Density (Ds) of the network is calculated as the number of existing connections divided by the maximum number of possible connections. Ds could be used as a direct measure of complexity of the Code. The more connections between the various parts of the Code exist, the more complex the Code is.

Average Clustering Coefficient. The Average Clustering Coefficient (AvCCo) is calculated as the arithmetic mean of the CCo of each Node of the network. Based on the definition of the CCo above, the AvCCo could be used to understand the flow and communication between neighboring and remote parts of the Code. High AvCCo would mean that there is limited flow and communication between the parts of the Code and that the Code has a strong hierarchical

---

[12] Art. 23.1, the Statement of the Principle of Priority, is the Article with the highest ClCe and thus the most central Article or the Article to which all other parts of the Code are closer to.

[13] The term 'Commission' does not enter the top-ten of parts of the Code in terms of WD. However, its BCe is, comparably, much higher, being the 6th higher in the network (only behind the terms 'author', 'taxon', 'publish', 'available name' and 'Code'). This means that is quite probable to encounter the Commission, not only directly in the Article of interest, but in its immediate connections. Also, Art. 8.1.3.2 ([...] a method that assures "widely accessible electronic copies with fixed content and layout") has low connections (WD = 8) but much higher BCe than expected, as it refers to the electronic publication. This is an important Article that one needs to understand when dealing with electronic-only publications.

structure. Low AvCCo would indicate that there is an open flow between the parts of the Code and an extended communication. In our case, I understand the AvCCo roughly as the amount of 'scrolling' or page-turning the user has to do[14]. With low AvCCo there are less complete networks and closed loops and even communication between remote parts. Thus, the user would need to scroll more or turn more pages to reach a conclusion.

Average Path Length and Diameter. The Average Path Length (PL) is calculated as the arithmetic mean of the shortest path length of all pairs of Nodes in the network. This would be a measure of the proximity of the answer to the user's question. One might ask: "*if I start from a certain part of the Code* (e.g., from a given Article or a Glossary item), *how many steps (or clicks, or page changes) I need to take to reach an answer?*" The PL could provide an estimate of that. The shortest path between two connected Nodes could, conceptually, represent a nomenclatural question: the user starts from a specific point of interest in the Code and following the connections of the *Code* moves from one part to another, until a conclusion is reached. The minimum steps needed to do that is the shortest path. The opposite, i.e., the maximum amount of steps (or clicks, or page changes) is given by the network Diameter. The Diameter (Dm) of the network is, simply put, the longest path in the network connecting any two Nodes.

[14] Some Articles are more autonomous than others. For example, Article 9 deals with "what does not constitute published work". This is basically an Article that can be used in autonomous way within the Code and has few connections with the rest of the Code (mostly with the terms of the Glossary). The AvCCo of its Nodes is 0.73, meaning practically that one of the most closed 'neighborhoods' of the *Code*. If a user 'lands' in Article 9, most probably he/she will find the answer therein. On the other hand, Article 23 on the "Principle of Priority" has an AvCCo of 0.25, because this Article is connected with most of the Code. If a user 'lands' in Article 23 looking for answer, it is quite probable that he/she would need to search on other parts of the Code as well.

Supplementary File 2:
Results of the Network Analysis

The first version of the NETICON ZOOLOGICON consists of 1,379 Nodes and 11,276 Edges; see Table 1 for network properties and Descriptors.

Table 1:
The network properties of the *Code*, after the inclusion of the 2012 Amendment, the Declaration 44 and the Declaration 45.

	Nodes	Edges	Average Degree	Average Weighted Degree	Density	Average Clustering Coefficient	Average Path Length	Network Diameter
With 2012 Amendment and Declaration 44 (i.e., online version of the *Code*)	1374	11243	16.354	25.836	0.012	0.34	2.899	7
Including Declaration 45	1379 (+ 5)	11276 (+ 33)	16.354 (0)	25.801 (– 0.035)	0.012 (0)	0.341 (+ 0.001)	2.898 (– 0.001)	7 (0)
The *Code* without the Glossary	1026	3059	5.963	18.517	0.006	0.464	5.109	12

The Code forms a giant component where all Nodes are connected to each other (see Giant Component filter in GEPHI). This practically means that there are no isolated parts of Code, and the user could start anywhere and reach any other part of the Code following the various connections. The number of connections of each Node (Degree) vary from 1 (the 10 Sub-Articles of Art. 58; some Examples; 5 Glossary items) to 273 (Glossary item: taxon). The top-34 Node in Degree connections are all Glossary items that include important concepts of the Code such as taxon, author, available name, species-group name, publish, species-group, species, species name, Commission; etc. The Article with the highest Degree is Art. 23.1 (Statement of the Principle of Priority), on position 35, when the Nodes are ranked according to their Degree. But

if we take into account the weight of the Edges (Weighted Degree), Art. 23.1 climbs up to position 7, as most of its 58 Edges are 'heavy'. In fact, Art. 23.1 has the greatest difference between Weighted and regular Degree, showing the importance of its connections.

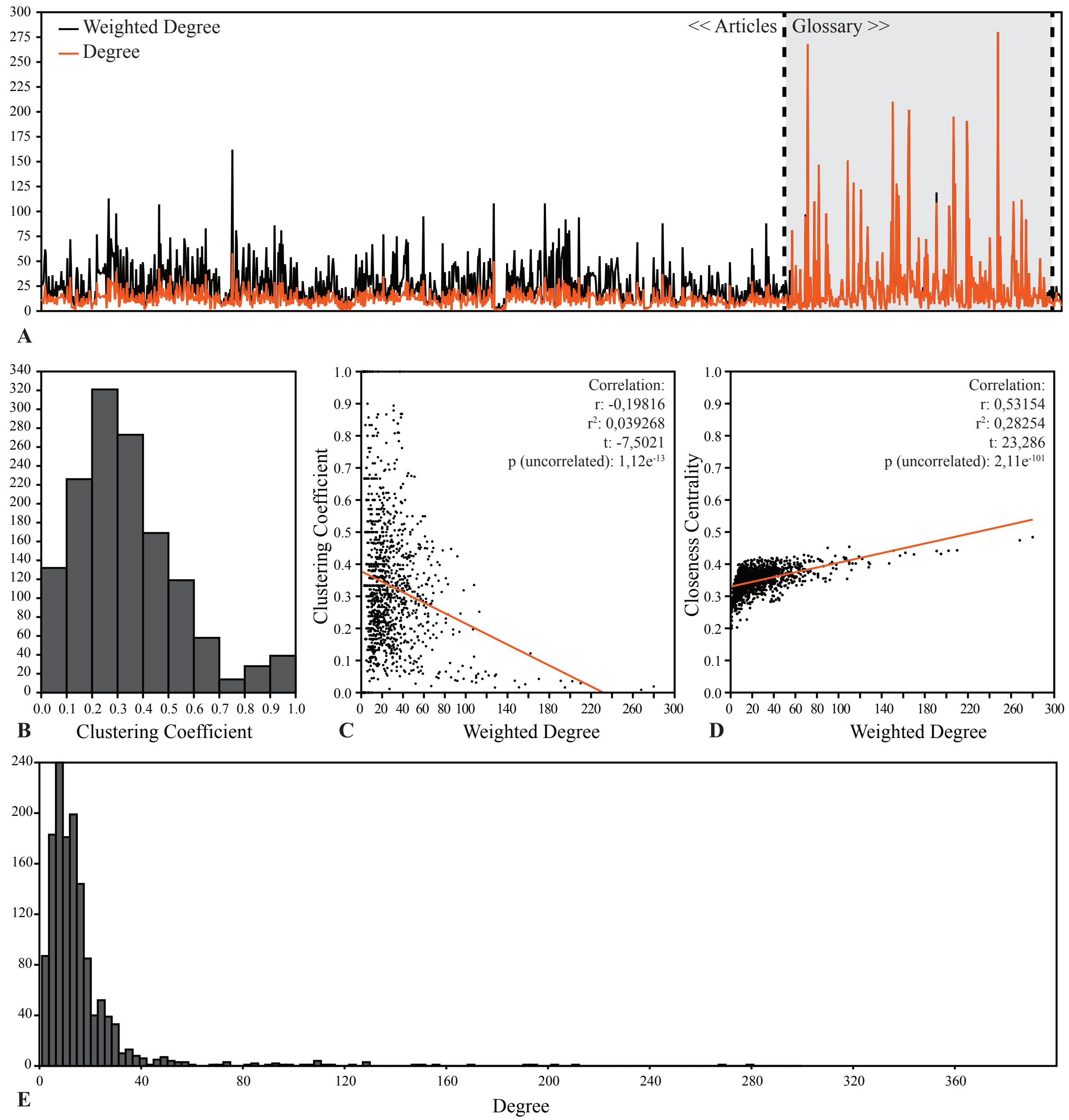

Figure 1: Diagrams of some of the descriptors of the network of the Code.

(A) Curves showing the variation of Degree and Weighted Degree across the Code. Articles are ordered according to their position in the current structure of the Code and a distinction between the main Articles and the Glossary is shown. (B) Histogram of the clustering of the Nodes. If the clustering of a given Node is 1.0 it means that this Node is connected to every other Node in its

neighborhood. (C) Linear correlation between the Weighted Degree and the Clustering Coefficient. (D) Linear correlation between the Weighted Degree and the Closeness Centrality. (E) Histogram depicting the distribution of Degree in the network.

Figure 1.A depicts the difference between Degree and Weighted Degree of each Node of the network. As it can be immediately seen, the Glossary items have many more connections than the other elements of the Code. As the connections between the Articles are heavier, they are responsible for the main structure of the Code, whereas the Glossary, through the connections of its items, would be important for binding the Articles together. Thus, the Glossary is an important part of the Code.

Figure 1.B represents the distribution of the Clustering Coefficient of the Nodes of the NETICON. As we can see, the majority of the Nodes have a clustering below 0.5. This means that there are very few parts of the Code that form close networks. The Clustering Coefficient of the various parts of the Code is negatively correlated with the Weighted Degree (Figure 1.C). That means that the most important parts of the Code are loosely connected with their surroundings and their conceptual influence extends to larger parts of the Code. Of course, the addition of the Glossary is partly responsible for this result.

Exceptions to this Rule include some Articles that are tightly connected with their Sub-Articles and show high Clustering Coefficient . For example, Art. 9 ('What does not constitute published work') forms a closed network, as the part "[…] *none of the following constitutes published work* […]" makes all its Sub-Articles to be connected to each other. In this case, each Node within Art. 9 is entirely connected with all the other Nodes in the neighborhood. Also, the majority of the parts of the Code that are connected to Art. 9 are mainly included in this Article or are Glossary items. This means that the way that Art. 9 is formulated and presented can be depicted in the form of continuous text without significant problems.

A look on the Closeness Centrality (Figure 1.D) reveals another interesting aspect of the Code: although there is a great variation on the amount of connections between the Articles, the Closeness Centrality varies less. Nodes with the highest Weighted Degree (e.g., taxon and author) have similar ClCe to Nodes with lower Weighted Degree, like Articles 23.1 and 23.6. This practically means that there are many central concepts in the Code and parts of the Code with fewer connections still could play a central part. More information on these metrics is given below, in the analysis of the Force Atlas 2 layout.

The Path Length is almost 3 steps (2.898). That practically means that the user of the Code, starting at any part of the text, would have to make only three steps (on average) to reach another part of the Code and get an answer to his/her question, of course following the network of the Code. Or, in other words, one needs to go through only four parts of the Code[15] (on average) to answer a specific question. The longest path of the network (its Diameter) is 7 steps. So, as a maximum, the user would have to go through eight parts of the Code to reach an answer[16].

Some other interesting conclusions come from the Betweeness Centrality (i.e., how often a Node appears on the shortest paths between Nodes in the network), as it could reveal some important Nodes as well. What is interesting is that, besides Nodes like 'taxon', 'author', 'publish' and 'available name', that are high on every category, the Betweeness Centrality of the 'Commission' (9th on D and 10th on Weighted Degree) and the 'Code' (20th on D and 21st on Weighted Degree) is high, placing them on the 5th and 6th position respectively.

The resulting network is a scale-free (Figure 1.E) network because the Degree follows a power-law distribution. The average Average Clustering Coefficient (0.34) and the small diameter (7) indicate that it is a small-world network. The Density of the network is low (0.012), meaning that there could be far more connections between the parts of the Code[17]. Therefore, this complex text forms actually a simple network, where the user is able to move from one part to the other with only few steps. This result would further emphasize that such a body of text cannot be fully used and understood as a continuous text. More insight on the structure of the network of the Code is presented on the following sections, where some layout algorithms are applied. The application of these algorithms helps visualizing the Code itself and actually use the network created herein.

Circular layout. The Circular layout of the Code is a visual representation of the current structure of the Code as a continuous text. The various parts of the Code are ordered around the circle according to their appearance in the text and their connections are shown. The main kinds of Edges that are visible are the Glossary (orange; 70% of the total), Relevant (brown; 14% of the

---

[15] Consider an example from an empirical point of view: these four parts could represent one Article, an Example/Recommendation and two Glossary items (e.g., I land on one Article, the Article leads me to its Recommendation and from there I go to the Glossary for the definition of two terms). This is quite efficient and I would say that it represents a kind of 'standard' interaction with the *Code*. There are, of course, many cases where the answer could be reached with a single step.

[16] Following the same example, this could mean two or three Articles, with one or two Examples/Recommendations and two or three Glossary terms.

[17] The maximum number of potential connections between the 1379 parts of the Code is 1,900,262.

total) and Article (black; 7% of the total); these types of Edges represent collectively 91% of the total connections of the Code. As it is immediately visible from Figure 1 of the main article, the connections between the Articles do not follow the continuous flow of the current structure of the Code. Of course, most Articles and Sub-Articles are connected with their nearby items, but there is a large amount of Edges that connect remote elements of the Code. If the current structure of the Code as a continuous text was adequate to present the connections between its articles, we shouldn't be able to see long lines connecting remote parts.

Force Atlas 2 layout. In order to depict the structure of the Code based on the number and weight of the connections I used the built-in Force Atlas 2 algorithm (Jacomy et al. 2014). The closer a Node is to the spatial center, the more connections it has with other Nodes. Nodes that are close together are more closely connected to each other. Similarly, Nodes that are distant from each other are loosely connected to each other. Therefore, this layout helps understanding better the structure of the Code. The different numbers on Figure 2 of the main article represent the different Articles of the Code and some of the main terms of the Glossary are shown as well. If the Glossary is excluded, the structure of the network is shown in Figure 2.A.

This layout algorithm helps understanding the conflicts in the current structure of the Code. Figure 3.B shows the various parts of the Code colored according to their placement in the various Chapters. In many cases, Articles belonging to the same Chapter are found closer together, forming their own hubs: Articles of the Chapter 1 (Zoological nomenclature; light green in Fig. 2.B) occupy the spatial center. Chapter 3 (Criteria of publication; navy blue in Fig. 2.B) and Chapter 5 (Date of publication; yellow-green in Fig. 2.B) occupy the bottom and bottom-left part of the network. Chapter 7 (Formation and treatment of names; pink in Fig. 3.B) occupies the right part of the network. Articles belonging to Chapters 14–16 (Types in family-, genus- and species-group; fuchsia, brown and orange colors respectively in Fig. 2.B) all form their own hubs and are, collectively, placed on the upper half of the network. Finally, a partly well-defined region of the NETICON ZOOLOGICON is the bottom to bottom-right corner, which contains most of the Articles of Chapters 17 (Commission; red in Fig. 2.B) and 18 (Regulations; light violet in Fig. 2.B). The exception refers to Article 81 (use of the plenary power) and several of its Sub-Articles, which tend to be placed towards the center of the network. This is because they deal, primarily, with the use of plenary power to set aside the Principle of Priority, whose related articles are situated in the central parts of the Code. The central part of the network is occupied by several Articles, terms and concepts, but the majority of them refers to the concepts of availability and

validity. These are the primary thematic areas of the NETICON ZOOLOGICON (inset in Fig. 2.B). The remaining Chapters occupy more central positions and there are cases where they are some that quite widespread. For example, Chapter 4 (Criteria of availability; yellow in Fig. 2.B) connects with parts in the entire Code—the importance of this Chapter is paramount, as it formulates the existence of available names in zoology.

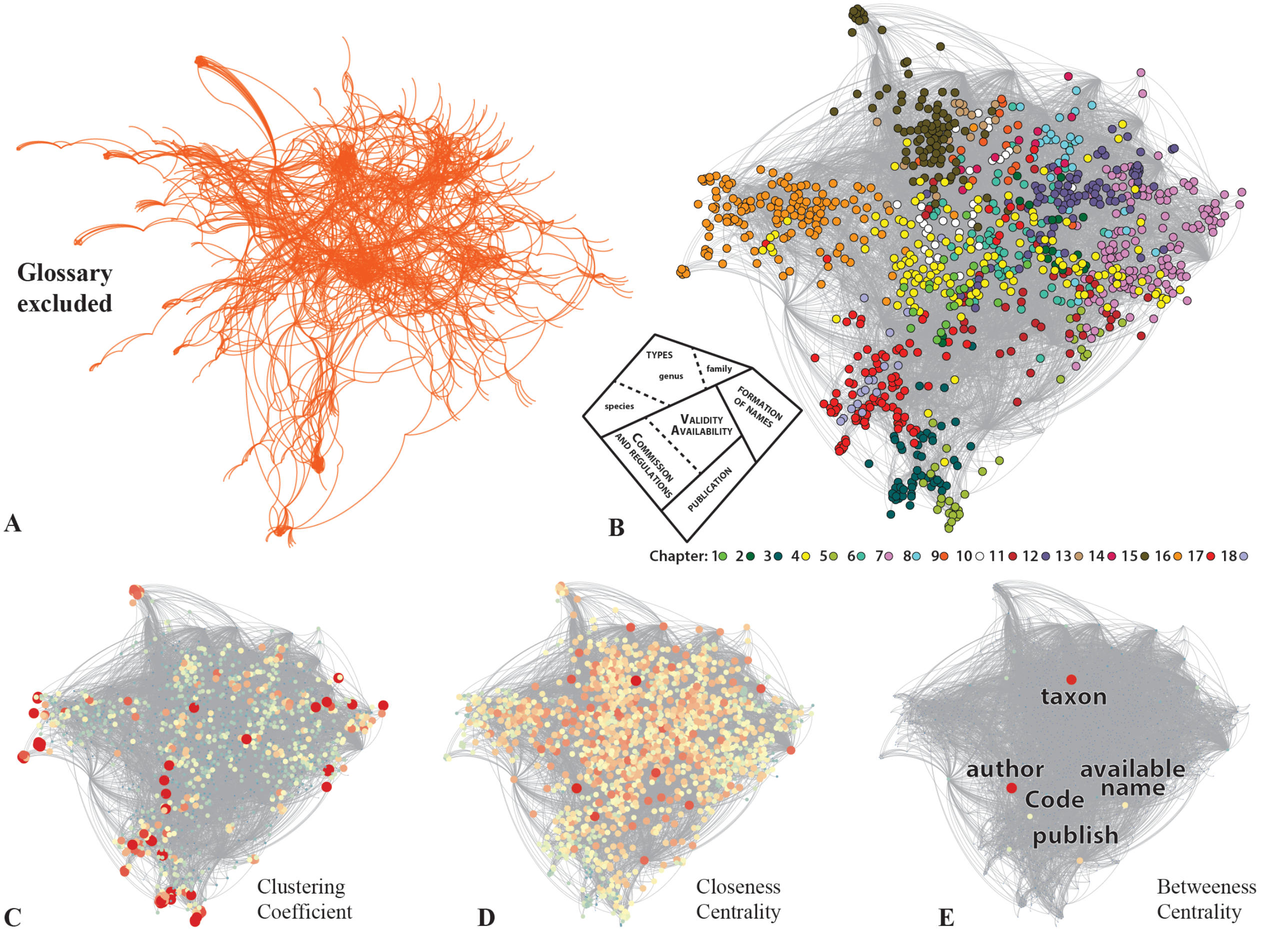

Figure 2: The analysis of the NETICON in Force Atlas 2 layout.

(A) the structure of the network if the Glossary is removed. (B) The same network, but the Articles are colored according to the Chapter they belong in. On its bottom left corner, a simplified diagram shows the thematic areas of the network. (C) The distribution of the Nodes according to their Clustering Coefficient. (D) The distribution of the Nodes according to their Closeness Centrality. (E) The distribution of the Nodes according to their Betweeness Centrality. In C–E, the magnitude of the corresponding metric for each Node is indicated by the size of the Node and its color (red: high, orange: middle, blue: low).

In Article-level, there are much more detailed observations to be made. For example, the Articles of Chapter 10 (Art. 45–49, on species-group taxa and their names; white color in Fig. 3.B) are placed in between Chapter 16 (types in the species group), Chapter 15 (types in the

genus group) and the Chapter 7 (on the formation and treatment of names). This is because the species is fundamental for the definition of a genus (i.e., type species) and the genus name affects the formation of the species name. For example, Article 48 is much closer to the Articles of the Chapter 7, than to the Articles of its own Chapter. As Article 48 is about the change of generic assignment and includes concepts like gender ending and combination of species-group names it is quite relevant with the concepts of Chapter 7. Another case is Article 21 (about the date of publication) that is much more closely related with Art. 7–9 (about publication criteria) than to its 'nearby' Articles in the Table of contents of the Code. Similarly, Articles 36, 43 and 46, as they speak about the same Principle (Principle of Coordination) are also close to each other even if they deal with a different rank. Although I deliberately added connections (Edges) that represent the current hierarchical structure of the Code, we can see that in several cases the continuous flow of the text of the Code is overridden by the contextual connections between the various elements.

This layout also helps understanding better the metrics, explained above, from a visual point of view. The distribution of the Clustering Coefficient of the Nodes (Figure 2.C) shows that the parts of the Code with high Clustering Coefficient are mainly distributed in the periphery of the network—from a network point of view, these would be the most isolated and remote parts of the Code, forming closed networks. These are some quite autonomous parts of the Code, like Article 9 (what does not constitute published work), Article 21 (determination of the publication date) and the Recommendations of Art. 72, 73, 74 and 75 (on the various kinds of type specimen and type locality). These would be parts of the Code that a reader can largely read in an autonomous way and be certain that the main message is captured. It is worthy to note that some remote parts of the Code could also appear in more central positions (i.e., those large, red, circles in the central part of the network in Figure 2.C). These are terms like sensu lato, Metazoa, auctorum and teratological specimen. Their central position (shown as well by their moderate ClCe) should not be mistaken as evidence of importance—instead, these terms are there because are directly connected with other important Nodes[18]. So, as it has been noted above, only one metric is not enough to estimate the importance of a Node. The Closeness Centrality, as it has been note above, shows that the Code contains several central concepts. As it is also visually seen in Figure 2.D, there is no single central point (i.e., large and red circles) in the network, but rather there are plenty of central points widespread in the network. There are, however, some concepts that are more important than others. As BCe shows (Figure 2.E), there are some terms that far exceed

[18] For example, *sensu lato* is connected with the terms Latin, *sensu* and *sensu stricto*, which occupy more central positions.

others in their tendency to appear during reading the Code: these are the terms author, taxon, publish, available name and Code. These are the terms that help us understand the Code, primarily, as an instrument that authors use to publish available names of taxa—but this is only the first step of the nomenclatural process.

The Code contains a figure that summarizes the "status of works, names and nomenclatural acts." This figure also depicts the basic stages that a name or a nomenclatural act pass through, since their publication until they are considered as valid. Dubois (2011; and references therein) took this a step further, by presenting four main stages of the nomenclatural process (availability, allocation, validity and correctness, registration) and by expanding this Summary figure in detail (Dubois 2011, Appendix). But what the network analysis tells us about this process? Clearly, terms that are relevant to the first part of the process (namely publication and availability) are signaled as the most important in the network (Fig. 3.E) in terms of Centrality. This does not mean, of course, that the other terms of the nomenclatural process are not also recovered as important. Allocation of an available name, which could be exemplified by the term 'name-bearing type' in the NETICON, is placed in the top-20 of the heaviest and most central Nodes. Validity of an available name, exemplified by the term 'valid name' in the NETICON, falls further behind the top-50 of the heaviest and most central Nodes. Registration, exemplified by the term 'register' in the NETICON, falls to the bottom of the Nodes when ranked according to their Degree and Centrality. This rank does not mean anything regarding the importance of these terms for the nomenclatural process; they are obviously indispensable and equally important terms and concept. But what this really means is that for the Code itself the first part of the nomenclatural process process, which regards the publication and availability, holds a much more extensive and central part of its legislative text. Excluding the part on the registration, I have mapped the First Degree Ego networks of the terms 'publish', 'available name', 'name-bearing type' and 'valid name' in the Force Atlas 2 layout (Figure 3.A) with different colors. These four subsets of the full network are also depicted isolated in the four corners of the Figure 3.B–E, each one with its own Force Atlas 2 layout. Whereas the First Degree connections of these four areas contain approximately 8 – 15% of the network, when extended to the Second Degree they exceed half of the network; thus, they are concepts that require in-depth knowledge of the Code. What it can be immediately noticed is that these four terms occupy mostly different areas of the network: publication (bluish green; Fig. 3.B) occupies mainly the bottom part of the network, availability (vermillon; Fig. 3.C) is placed centrally and just above publication, name allocation (reddish

purple; Fig. 3.D) occupies the upper left part, and validity (sky blue; Fig. 3.E) the upper right part of the network.

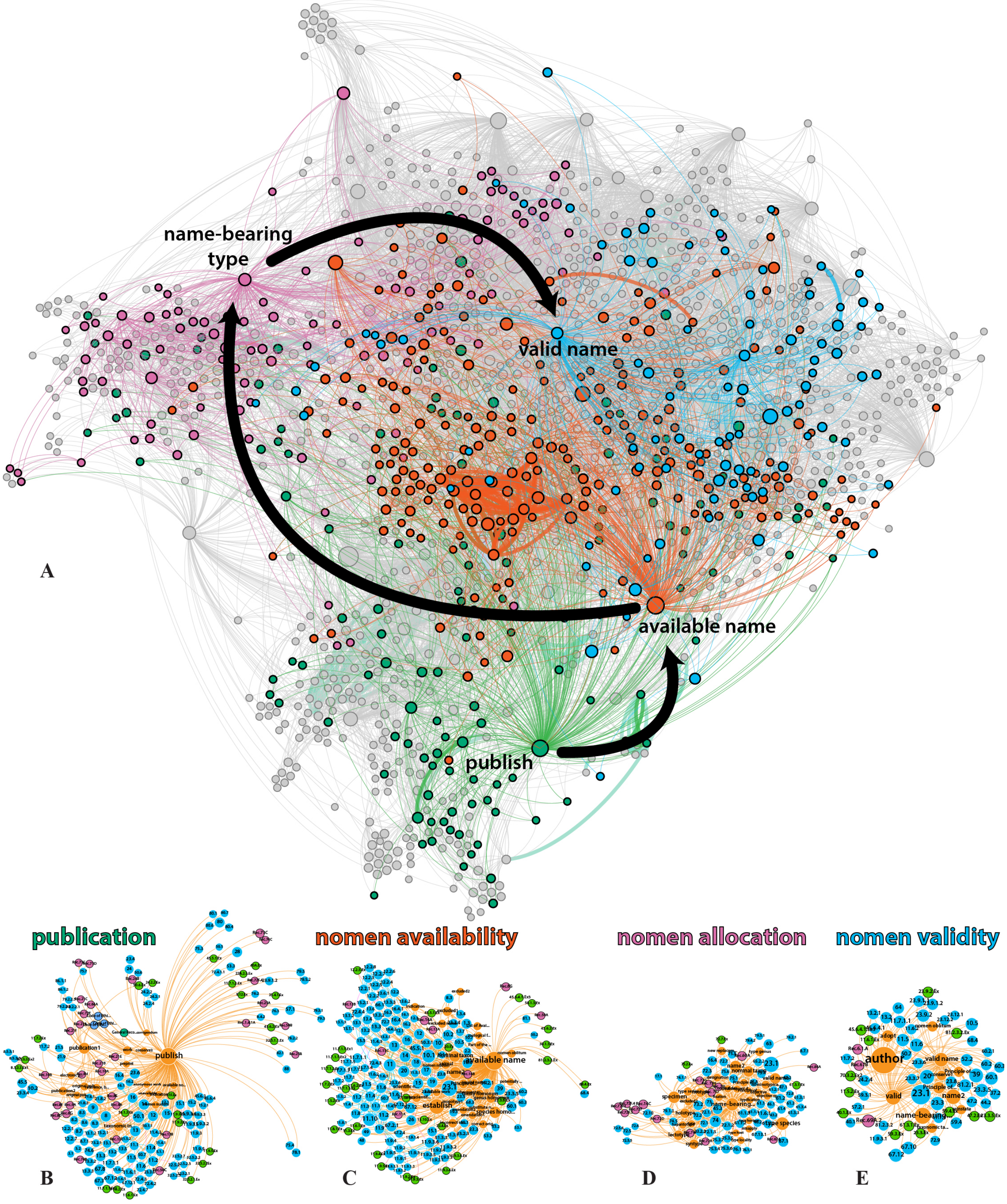

Figure 3: **The nomenclatural process depicted in the NETICON.**

A, the main stages of the nomenclatural process are mapped with different colors in the network, by using a central term and its 1st Degree connections (Ego networks in Gephi). Each subset is

separated in its own Force Atlas 2 network. B, the nomenclatural process starts with the publication of a name (bluish green). C, publication creates available names (vermillon). D, these names are allocated to taxa with name-bearing types (reddish purple). E, then, names enter nomenclature to compete for validity (sky blue).

Of course, all these four concepts contain directly connected terms that are placed in remote locations and within the other concepts. So, although the NETICON shows up that the different parts of the nomenclatural process occupy primarily distinct areas in the Code, there is some inevitable mixing between the steps and their boundaries are not so easily defined in some cases. Dubois (2011; and references therein) has also called these stages as 'storeys' or 'floors' of the 'nomenclatural house', criticizing correctly that the current structure of the Code does not accurately highlights this fundamental process. The results of the NETICON strongly concur with Dubois's views, as the nomenclatural process is recovered in the NETICON (Figure 3). The network analysis also allows observing the interconnections and overlap between these four stages. Still, the first part of the process, namely the publication and availability of names, clearly overshadows the rest in the text of the Code. Clearly, without the first floor we would have no building at all.

The importance of the Glossary. Article 89 dictates that our interpretation of the Code should be based on the meanings of words and expression as explained in the Glossary. In the Preamble of the Code it is also stated that "[…] *this Preamble and the Glossary are integral parts of the Code's provisions*". These references alone should be enough to express the importance of the Glossary; but I am not sure if this is evident to many users. For example, there are terms that are almost only mentioned in the Glossary. The term '*nomen nudum*' is only mentioned in the main text of the Code in the Example of Art. 51.2 and nowhere else in the Code! But on the other hand, there are several Articles in the Code that are directly and indirectly relevant to this term. The definition of a *nomen nudum* is given as "[a] ***Latin*** *term referring to a name that, if* ***published*** *before* ***1931****, fails to conform to Article* ***12****; or, if published after* ***1930****, fails to conform to Article* ***13****. A nomen nudum is not an* ***available name*** *and therefore the same name may be made available later for the same or a different concept; in such a case it would take* ***authorship*** *and* ***date [Art. 50, 21]*** *from that* ***act*** *of* ***establishment****, not from any earlier publication as a nomen nudum*" (ICZN 1999, Glossary; emphasis added with boldface). The words in boldface indicate some of the terms, articles and concepts that are directly connected with the *nomen*

nudum term. Besides those, we can certainly find more that are implied to be directly connected with this term. For example, the definition of '*nomen nudum*' includes the words "not an available name" which implies that a *nomen nudum* is not only connected with the concept of the "available name" but also with that of "unavailable name". In this network, a number of direct and indirect, Edges connect the term "*nomen nudum*" with the rest of the Code. In total, the term '*nomen nudum*' is directly connected with at least 16 different parts of the Code (Glossary: author, nomenclatural act, date of publication, establish, Latin, available name, unavailable name, publish; Articles 12, 13, 21, 50; Example 51F; 1930 and 1931 dates) and through them to at least 674 more Nodes (which translates to almost 50% of the network). In other words, a term that appears only once in the text of the Code (and only in an example) has, via its definition in the Glossary, a notable direct and indirect presence in the entire Code.

Extending this logic, it is easy to grasp that the influence of the Glossary items extends to the entire Code. Visually, this is evident in Figures 2 and 3, where the edges connecting the Glossary terms (orange color) dominate the network of the Code. Alternatively, the Glossary Edges represent 70% of the total connections between the elements of the Code. However, I deliberately added the minimum weight (1.0) to those edges (see Methods). As such, the Glossary items are not responsible for the main structure of the Code.

How would the Code be without the Glossary? If the Glossary Nodes (and their Edges) are removed from the network, the entire structure remains practically the same (see Figure 4.A), but the consequences are more evident from the interpretation of the various metrics (Table 1). A Code without the Glossary would be a much simpler network (Density = 0.006) and a network with less flow (Average Clustering Coefficient = 0.464). The absence of the Glossary, however, would actually require more 'steps' by the user to reach a conclusion, both on average (Average Path Length = 5.109) and as a maximum (Diameter = 12). Thus, a Code without the Glossary would be a less efficient structure. The influence of the Glossary items extends to the entirety of the Code and is responsible for the general connectedness of the network. In other words, the Glossary items should be seen as the paths that bind the various elements of the Code together. It is through the Glossary that we can fully understand the connections between the various concepts and elements of the Code and to estimate the potential effect of changes and amendments.

The same cannot be said for the other non-legislative parts of the Code (i.e., Recommendations, Examples, Appendices, Preamble). Their removal from the Neticon does alter significantly the structure (Average Clustering Coefficient = 0.358, Average Path Length =

2.892, Density = 0.015, Diameter = 7). This is expectable as these parts (and especially the Recommendations and Examples) are mostly extensions of the Articles. Thus, the user would reach the same conclusion without these parts, but of course these parts add more explanatory power to the Code.

Supplementary File 3:
Re-structuring the presentation of the Code

The objective is to create an easy-to-comprehend text that introduces the reader to the topic of "Greek". The following text is an example on how the current text of the Code could be re-organized to form a text, following the connections and the structure of the network. Parts of the text that are underlined are directly quoted from the Code.

Use of Greek and Ancient Greek words under the Code

Important! For the purposes of the Code, the use of Greek words to form new names strictly refers to Ancient Greek words, as they appear in standard Ancient Greek dictionaries. [Glossary]

Ancient Greek is one of the languages that can be used to form available names. In general, a name may be a word in or derived from Latin, Greek or any other language (even one with no alphabet), or be formed from such a word. It may be an arbitrary combination of letters providing this is formed to be used as a word. [Art. 11.3]
Example. *Toxostoma* and *brachyrhynchos* from the Greek.

In some cases, the derivation of a name from the Ancient Greek is assumed. For example, when the spelling of a scientific name, or of the final component word of a compound name [Art. 31.1], is the same as an Ancient Greek word, that name or that component is deemed to be a word in the relevant language unless the author states otherwise when making the name available.

As a scientific name must, when first published, have been spelled only in the 26 letters of the Latin alphabet (taken to include the letters j, k, w and y) [Art. 11.2], Ancient Greek words should be transformed into Latin.

There are two ways to do that:

1) To transliterate the Ancient Greek word, which is the replacement of the letters of one alphabet by equivalent letters of another. [Glossary]

2) To latinize the Ancient Greek word, which means to give Latin form and characteristics (including a Latin ending or a Latin suffix) to any word which is not Latin. [Glossary]

Although Ancient Greek words can be used to form names at any rank, it is important to start from the genus-group names (i.e., genera and subgenera).

First of all, a genus-group name must be, or be treated as, a singular noun in the nominative case [Art. 11.8].

When forming genus-group names from Ancient Greek words, it is important to pay attention to the gender of the genus-group name. The gender is a grammatical property (masculine, feminine or neuter) that affects the way in which Latin or latinized adjectival or participial species-group names are to be spelled [Glossary, part].

The gender of the name is indicated by its gender ending, which is defined as the letters at the end of a genus-group name [Glossary]. Therefore, the gender of names formed from Ancient Greek words is determined as follows, based on whether the name was formed via transliteration or latinization of the Ancient Greek word [Art. 30.1]:

1) A genus-group name that is or ends in an Ancient Greek word transliterated into Latin without other changes takes the gender given for that word in standard Ancient Greek dictionaries [Art. 30.1.2]

Examples. Ancient Greek nouns transliterated without change into Latin as the whole or part of a name: *Ichthyornis*, ending in -*ornis* (ornis), is masculine; *Lepas* (lepas) is feminine; *Diadema* (diadema) is neuter. Names ending in -*caris* (caris), -*gaster* (gaster), -*lepis* (lepis), or -*opsis* (opsis) are feminine; names ending in -*ceras* (keras), -*nema* (nema), -*soma* (soma), -*stigma* (stigma), or -*stoma* (stoma) are neuter.

2) A genus-group name that is an Ancient Greek word latinized with change of ending, or with a Latin or latinized suffix, takes the gender normally appropriate to the changed ending or the Latin suffix [Art. 30.1.3]

Examples. Names with the Latin gender ending -*us*, latinized from the Ancient Greek endings -os (masculine or feminine), -e (feminine), -a (neuter) or -on (neuter), are masculine: e.g. -*cephalus* (kephale), -*cheilus* and -*chilus* (cheilos), -*crinus* (krinon), -*echinus* (echinos), -

gnathus (gnathos), -*rhamphus* (rhamphos), -*rhynchus* (rhynchos), -*somus* (soma), -*stethus* (stethos), and -*stomus* (stoma). Names ending in the Latin gender ending -*a*, latinized from the Ancient Greek ending -on are feminine, e.g. -*metopa* (metopon). Names derived from the Ancient Greek -keras (neuter) may have the ending -*cerus* (masculine) or -*cera* (feminine), although simple transliteration of the Ancient Greek ending as -*ceras* retains the neuter gender; *Phorella* (feminine) is derived from the Ancient Greek word phor (a robber, masculine) and the Latin diminutive suffix -*ella* (feminine); *Scatella*, feminine, is derived from skatos (neuter) and the Latin suffix -*ella* (feminine); *Doridunculus* (masculine) from Doris, Ancient Greek, the name of a sea godess (feminine), and -*unculus* a Latin suffix (masculine).

The following exceptions apply:

If the author states when establishing the name that it is not formed from, or is not treated as, a Latin or Ancient Greek word [Art. 26], the gender is determined as though the name is an arbitrary combination of letters (Article 30.2.2) [Art. 30.1.4.1]

See also Art. 30.2 for the gender of names formed from words that are neither Latin nor Ancient Greek.

It is recommended that the gender is to be made self-evident. So that the gender of new genus-group names is self-evident, authors, when forming new names based on words that are not Latin or Ancient Greek and stating their genders, are advised to choose genders for them appropriate to their endings. [Recommendation 30B]

Knowing the gender of the name formed by an Ancient Greek word is important in zoological nomenclature, since the gender form of such a species-group name must agree with the gender of the generic name with which it is combined [Glossary, part].

This is mandatory when the species-group name, is or ends in a Latin or latinized adjective or participle in the nominative singular [Art. 31.2]. In that case, its gender ending—the letters at the end of the latinized Ancient Greek adjectival species-group name—must agree in gender form with the gender of the generic name with which the species-group name is combined [Glossary]. For exceptions, please see Art. 32.2.1–3.

Supplementary File 4:

How to use the NETICON

My intention is to provide this network in open access, for all the users that are interested in the *Code*. The access and use of the network can be done in two ways, first with an online version that allows some basic navigation and with the full network through GEPHI. See the webpage at <http://staff.mef.org.ar/en/researchers/evangelos-vlachos/iczn-network> for further information, including videos demonstrating how to use these networks, and the OSF repository (osf.io/rjq7n).

Html versions of the networks

On the above-mentioned links the user can find the final html network (current version), on a Force Atlas 2 layout, exported with the plugin SIGMAEXPORTER 0.9.0 (see Methods). An alternative is to download the offline version of the html network, which a zipped folder named "network" and contains all the relevant files. In this case, the user should simply open the file "index.html" on any web browser other than Google Chrome; note that this html page does not work well on tablets and smartphones. The first thing that the user sees is the entire network as in the figures herein. Different colors represent the different kinds of Nodes and Edges as defined herein. Different sizes of the Nodes refer to their relative number of connections. Next, I will briefly explain how to use and navigate. First, the user can place the cursor above any Node. Then this Node is highlighted, along with its First-Degree connections and the name of the Node appears; the remaining parts of the network are shaded. If the user clicks on a Node, then only this Node and its First-Degree connections appear, whereas the rest disappear. Also, a new window appears on the right that contains some basic network information of this Node, its contents (e.g., the content of that selected Article) and below all its connections. Each of these connections can be subsequently selected and then the user moves on the following Node of interest. Alternatively, the user could use the search bar on the left and by entering a term (e.g., '1.1' referring to Article 1.1, or 'holotype' referring to the relevant Glossary item) this particular Node is selected. At any point, the user can select the 'return to the full network' option on the top right of the pop-up menu and see the entire network once again. This is a locked network and the user can only navigate within.

This tool could be used to cover numerous basic needs as a companion to the Code. The user can use this network to read the various Articles according to their structural connections with few clicks, without having to scroll down (online version) or turn (printed version) numerous

pages. By using the network version instead of the entire Code, the user has a feeling of the overall importance of his/her query. For example if the user is interested in the term 'holotype', the network returns 48 primary connections of this term through the Code. Assuming that most of these primary connections could (or will) have some other secondary, but directly relevant to the initial query (e.g., the term holotype is connected with the various 'types'), the user immediately gets that he/she has to review some tens or hundreds of Articles at least for the full comprehension of the meaning and use of 'holotype'. Besides knowing that in a vague way (probably every zoologist understands the importance of holotypes for zoological nomenclature), this tool gives exactly which parts of the Code ones needs to read. Instead, if one is interested on the term 'mimeographic' finds only one connection with Art. 9.2—in this case ones needs basically to read Article 9 (what does not constitutes published work).

Besides daily use, I suppose that this tool could be extremely useful for communicating and teaching the Code. Seeing the Code as a network, is much more inviting than a complete volume of hundreds of pages, or perhaps a webpage that follows this structure. As a network, the size and complexity of the Code seems more manageable to the average zoologist or to a student. After spending few hours with this network trying to read through the Code, the user can return to the original text with a different mentality or renewed interest, perhaps also without fear or concerns about possible connections that he/she might ignore. For a student, this could also be an ideal starting point to get familiar with the Code. With a series of designed exercises, the student can use the network to solve a question, without getting discouraged by the overwhelming size of the Code itself.

Original network

Besides the web version of the network, I also provide on the same link the original network file with all raw data. This file can be opened with the free software GEPHI. As GEPHI is quite user-friendly and is relatively easy to learn through the available documentation, I am certain that users that are interested in working more with this network will find this option quite interesting. GEPHI has three main windows: the 'Data Laboratory', a spreadsheet-like tab which contains all network information, the Nodes and the Edges; the 'Overview' that shows the network, where the user could experiment with the layout of the network manually, with the numerous algorithms therein, calculate statistics and apply filters to the network; and the 'Preview', where the user can make the final layout adjustments (colors, fonts) before exporting the network.

First of all, the user is invited to make all the necessary changes to the current version of the network. These could be errors, missing connections or additions to the existing network (in this case you can also contact me, because I intend to provide regular updates/versions to the network). Also, the user can also use the various algorithms to create various layouts of the network. Perhaps the most powerful set of tools in GEPHI is the set of filters. The user can apply various filters to the Nodes and/or edges to isolate specific parts of the network. What I find of particular use is the so-called 'Ego Network', where the user selects a Node and a depth of connections (i.e., the Degree of connections from the selected Node). This filter could be used in several cases, for example when someone wants to investigate the effect of changing/editing/removing parts of the Code. In this case, the use can select the particular Node (Article) and see the effect that it has on the entire Code (see case studies herein). Also, the filters not only isolate the Nodes and Edges of the network visually in the 'Overview' tab, but also make a selection of the relevant Nodes and Edges in the 'Data Laboratory' tab. So, the user could use the filters to make a selection of the relevant Nodes of a desired query (e.g., 'holotype') and then export the selected Nodes as a spreadsheet and text file, containing all the relevant information of the Code.

References

Anonymous [International Commission on Zoological Nomenclature]. 1999. *International code of zoological nomenclature*. 'Fourth edition'. London: (International Trust for zoological Nomenclature).

Bastian M, Heymann S, Jacomy M. 2009. Gephi: an open source software for exploring and manipulating networks. *Proceedings of the third International ICWSM Conference* 8:361–362.

Dubois A. 2011. The *International Code of Zoological Nomenclature* must be drastically improved before it is too late. *Bionomina* 2:1–104.

Esteve Altava B. 2013. *Structural analysis of network models in tetrapod skulls. Evolutionary trends and structural constraints in morphological complexity, integration and modularity*. Valencia: University of Valencia.
