# Supplementary figures and images for: Introducing a new tool to navigate, understand and use International Codes of Nomenclature
Source: PeerJ. 2019 Nov 25;7:e8127. doi: 10.7717/peerj.8127 (PMC6882414; doi:10.7717/peerj.8127)

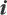

Supplement: Supplemental Information 3 — This zipped file contains a folder with the live network for offline use. Please unzip the file, and locate the file called ”index.html”. Please open it with any browser exc. Google Chrome. You will get a webpage with the live network, for navigation purposes. This version of the network cannot be edited. Exported with the built-in plugin in Gephi, developed by the InteractiveVis project of the Oxford Internet Institute. [file peerj-07-8127-s003.zip › network/images/info.png]

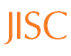

Supplement: Supplemental Information 3 — This zipped file contains a folder with the live network for offline use. Please unzip the file, and locate the file called ”index.html”. Please open it with any browser exc. Google Chrome. You will get a webpage with the live network, for navigation purposes. This version of the network cannot be edited. Exported with the built-in plugin in Gephi, developed by the InteractiveVis project of the Oxford Internet Institute. [file peerj-07-8127-s003.zip › network/images/jisc-logo-small.png]

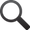

Supplement: Supplemental Information 3 — This zipped file contains a folder with the live network for offline use. Please unzip the file, and locate the file called ”index.html”. Please open it with any browser exc. Google Chrome. You will get a webpage with the live network, for navigation purposes. This version of the network cannot be edited. Exported with the built-in plugin in Gephi, developed by the InteractiveVis project of the Oxford Internet Institute. [file peerj-07-8127-s003.zip › network/images/zoom_reset.png]

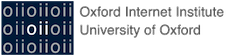

Supplement: Supplemental Information 3 — This zipped file contains a folder with the live network for offline use. Please unzip the file, and locate the file called ”index.html”. Please open it with any browser exc. Google Chrome. You will get a webpage with the live network, for navigation purposes. This version of the network cannot be edited. Exported with the built-in plugin in Gephi, developed by the InteractiveVis project of the Oxford Internet Institute. [file peerj-07-8127-s003.zip › network/images/oii_text.png]

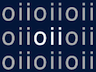

Supplement: Supplemental Information 3 — This zipped file contains a folder with the live network for offline use. Please unzip the file, and locate the file called ”index.html”. Please open it with any browser exc. Google Chrome. You will get a webpage with the live network, for navigation purposes. This version of the network cannot be edited. Exported with the built-in plugin in Gephi, developed by the InteractiveVis project of the Oxford Internet Institute. [file peerj-07-8127-s003.zip › network/images/oii.png]

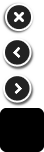

Supplement: Supplemental Information 3 — This zipped file contains a folder with the live network for offline use. Please unzip the file, and locate the file called ”index.html”. Please open it with any browser exc. Google Chrome. You will get a webpage with the live network, for navigation purposes. This version of the network cannot be edited. Exported with the built-in plugin in Gephi, developed by the InteractiveVis project of the Oxford Internet Institute. [file peerj-07-8127-s003.zip › network/images/fancybox_sprite.png]

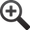

Supplement: Supplemental Information 3 — This zipped file contains a folder with the live network for offline use. Please unzip the file, and locate the file called ”index.html”. Please open it with any browser exc. Google Chrome. You will get a webpage with the live network, for navigation purposes. This version of the network cannot be edited. Exported with the built-in plugin in Gephi, developed by the InteractiveVis project of the Oxford Internet Institute. [file peerj-07-8127-s003.zip › network/images/zoom_in.png]

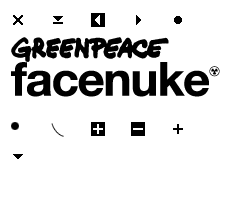

Supplement: Supplemental Information 3 — This zipped file contains a folder with the live network for offline use. Please unzip the file, and locate the file called ”index.html”. Please open it with any browser exc. Google Chrome. You will get a webpage with the live network, for navigation purposes. This version of the network cannot be edited. Exported with the built-in plugin in Gephi, developed by the InteractiveVis project of the Oxford Internet Institute. [file peerj-07-8127-s003.zip › network/images/sprite.png]

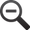

Supplement: Supplemental Information 3 — This zipped file contains a folder with the live network for offline use. Please unzip the file, and locate the file called ”index.html”. Please open it with any browser exc. Google Chrome. You will get a webpage with the live network, for navigation purposes. This version of the network cannot be edited. Exported with the built-in plugin in Gephi, developed by the InteractiveVis project of the Oxford Internet Institute. [file peerj-07-8127-s003.zip › network/images/zoom_out.png]

Supplement: Supplemental Information 3 — This zipped file contains a folder with the live network for offline use. Please unzip the file, and locate the file called ”index.html”. Please open it with any browser exc. Google Chrome. You will get a webpage with the live network, for navigation purposes. This version of the network cannot be edited. Exported with the built-in plugin in Gephi, developed by the InteractiveVis project of the Oxford Internet Institute. [file peerj-07-8127-s003.zip › network/images/blank.gif]

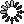

Supplement: Supplemental Information 3 — This zipped file contains a folder with the live network for offline use. Please unzip the file, and locate the file called ”index.html”. Please open it with any browser exc. Google Chrome. You will get a webpage with the live network, for navigation purposes. This version of the network cannot be edited. Exported with the built-in plugin in Gephi, developed by the InteractiveVis project of the Oxford Internet Institute. [file peerj-07-8127-s003.zip › network/images/fancybox_loading.gif]

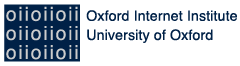

Supplement: Supplemental Information 3 — This zipped file contains a folder with the live network for offline use. Please unzip the file, and locate the file called ”index.html”. Please open it with any browser exc. Google Chrome. You will get a webpage with the live network, for navigation purposes. This version of the network cannot be edited. Exported with the built-in plugin in Gephi, developed by the InteractiveVis project of the Oxford Internet Institute. [file peerj-07-8127-s003.zip › network/images/oii_brand.png]

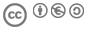

Supplement: Supplemental Information 3 — This zipped file contains a folder with the live network for offline use. Please unzip the file, and locate the file called ”index.html”. Please open it with any browser exc. Google Chrome. You will get a webpage with the live network, for navigation purposes. This version of the network cannot be edited. Exported with the built-in plugin in Gephi, developed by the InteractiveVis project of the Oxford Internet Institute. [file peerj-07-8127-s003.zip › network/images/CC.png]

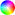

Supplement: Supplemental Information 3 — This zipped file contains a folder with the live network for offline use. Please unzip the file, and locate the file called ”index.html”. Please open it with any browser exc. Google Chrome. You will get a webpage with the live network, for navigation purposes. This version of the network cannot be edited. Exported with the built-in plugin in Gephi, developed by the InteractiveVis project of the Oxford Internet Institute. [file peerj-07-8127-s003.zip › network/images/rainbow.png]

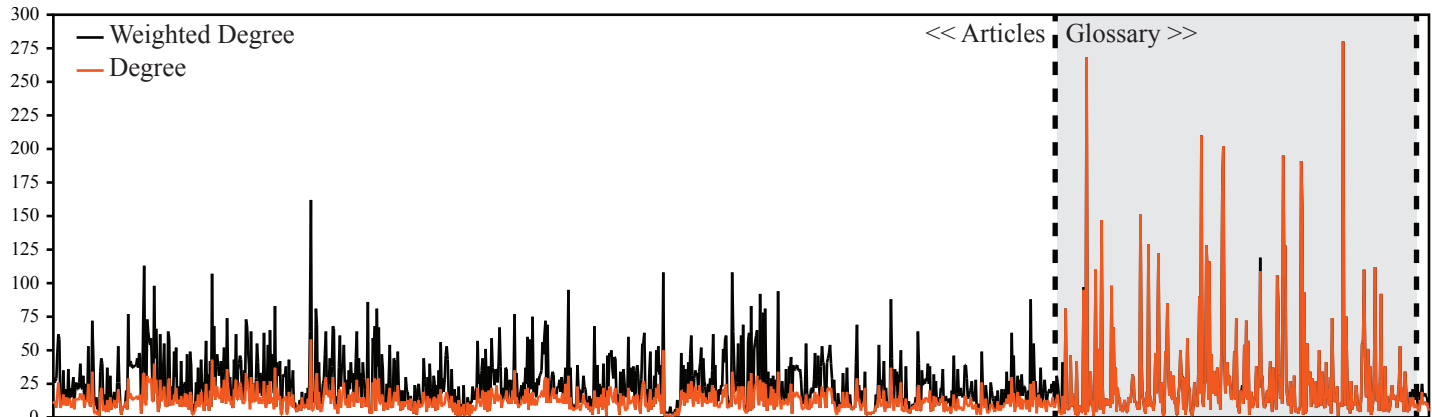

A

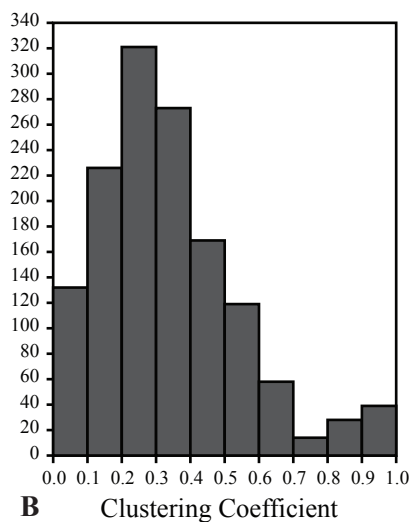

B

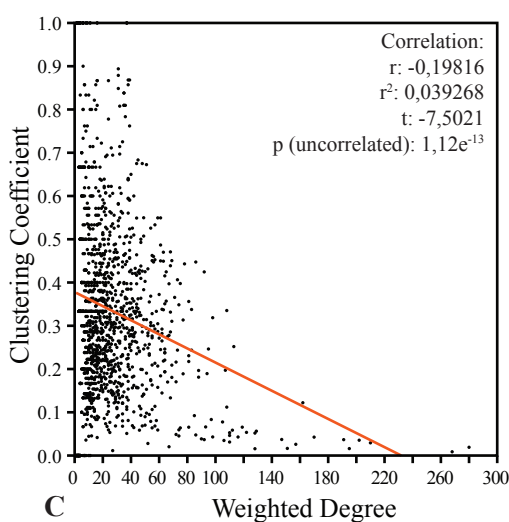

C

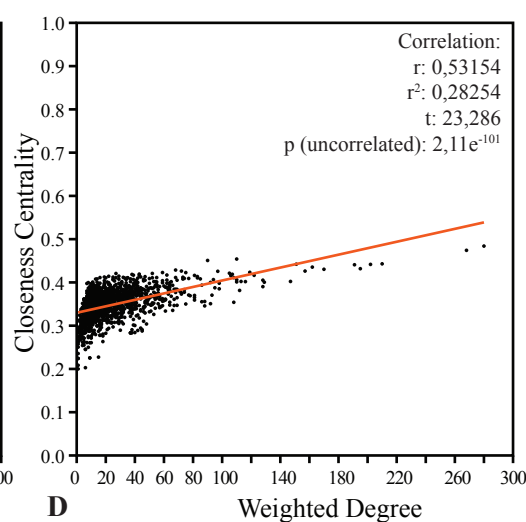

D

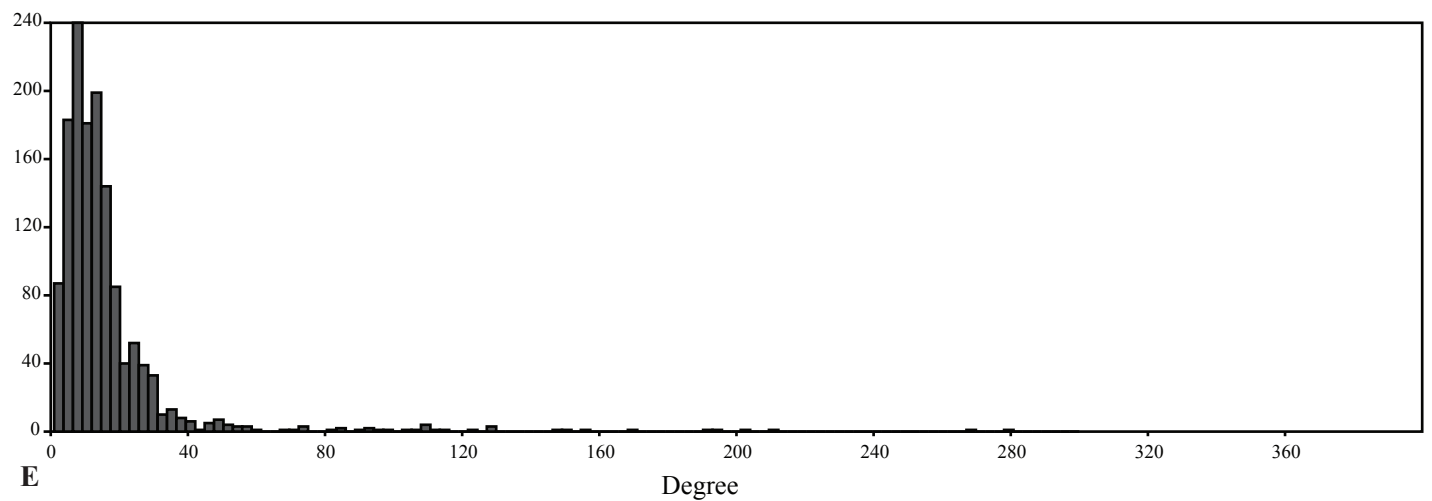

E

Supplement: Figure S1 — (A) Curves showing the variation of Degree and Weighted Degree across the Code. Articles are ordered according to their position in the current structure of the Code and a distinction between the main Articles and the Glossary is shown. (B) Histogram of the clustering of the Nodes. If the clustering of a given Node is 1.0 it means that this Node is connected to every other Node in its neighborhood. (C) Linear correlation between the Weighted Degree and the Clustering Coefficient. (D) Linear correlation between the Weighted Degree and the Closeness Centrality. (E) Histogram depicting the distribution of Degree in the network. [file peerj-07-8127-s004.pdf]

Glossary  
excluded

A

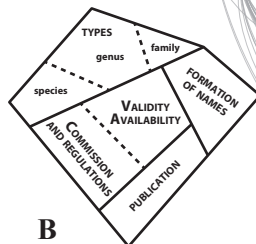

B

Chapter: 1 ● 2 ● 3 ● 4 ● 5 ● 6 ● 7 ● 8 ● 9 ● 10 ○ 11 ● 12 ● 13 ● 14 ● 15 ● 16 ● 17 ● 18 ●

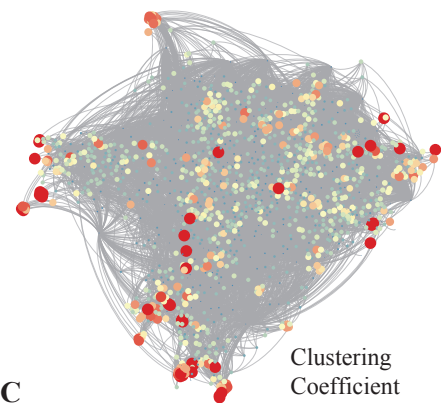

C

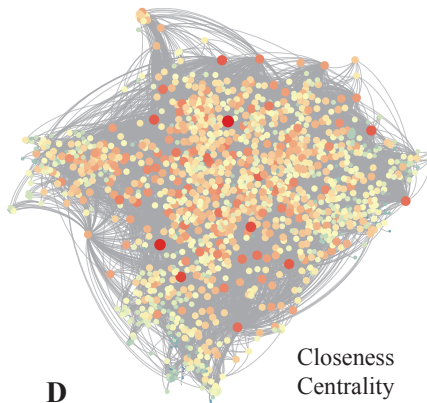

D

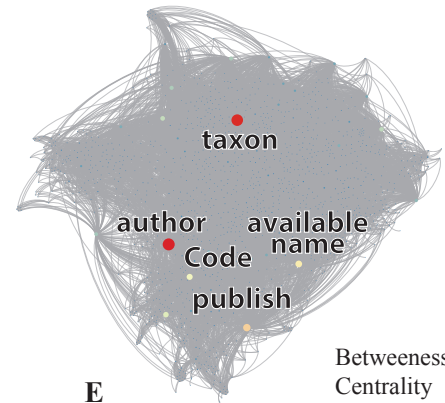

E

Supplement: Figure S2 — (A) the structure of the network if the Glossary is removed. (B) The same network, but the Articles are colored according to the Chapter they belong in. On its bottom left corner, a simplified diagram shows the thematic areas of the network. (C) The distribution of the Nodes according to their Clustering Coefficient. (D) The distribution of the Nodes according to their Closeness Centrality. (E) The distribution of the Nodes according to their Betweeness Centrality. In C–E, the magnitude of the corresponding metric for each Node is indicated by the size of the Node and its color (red: high, orange: middle, blue: low). [file peerj-07-8127-s005.pdf]

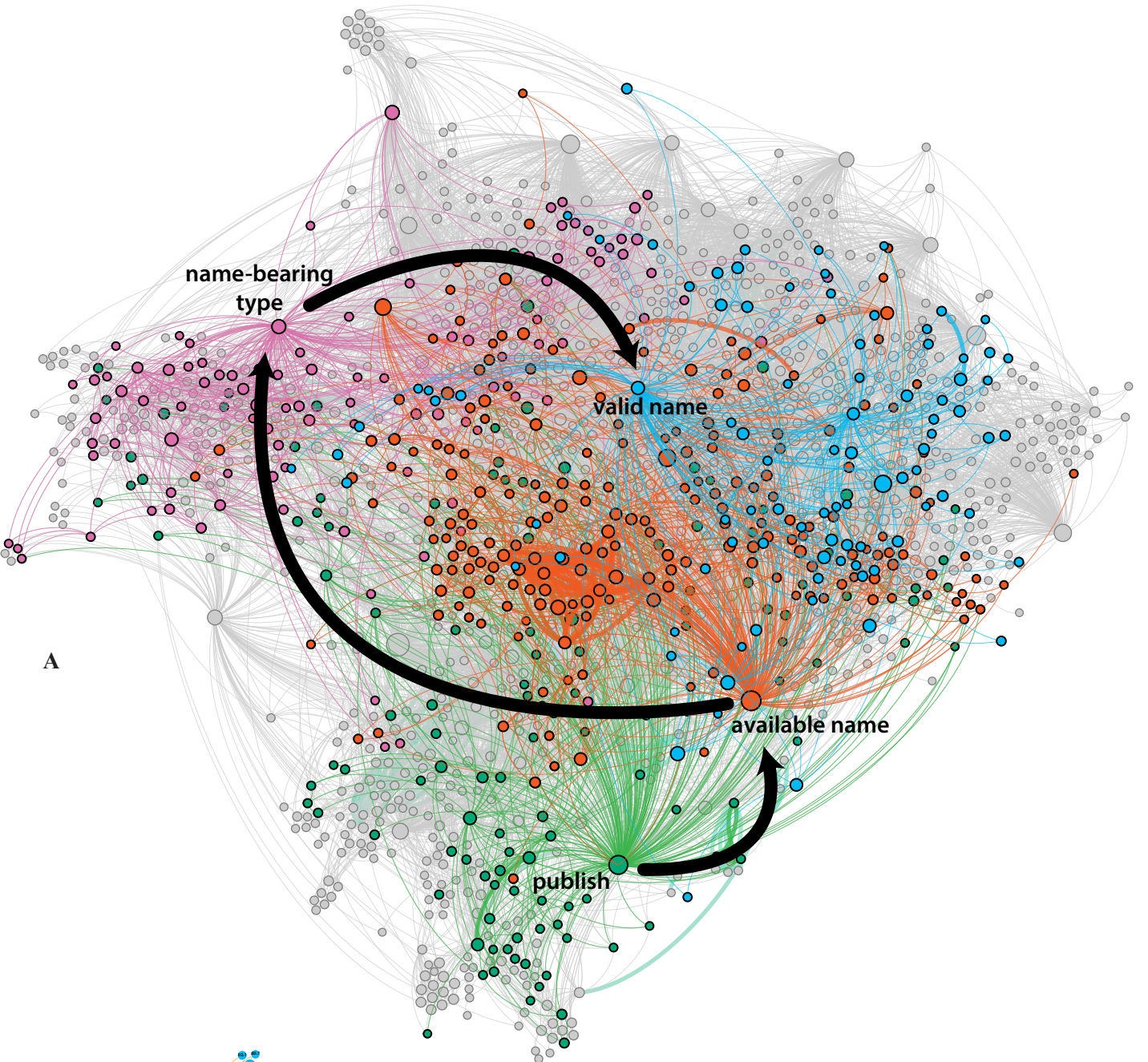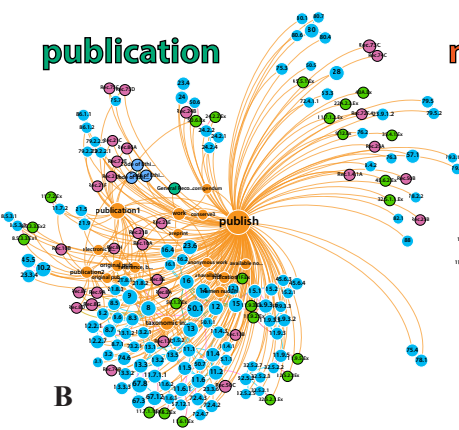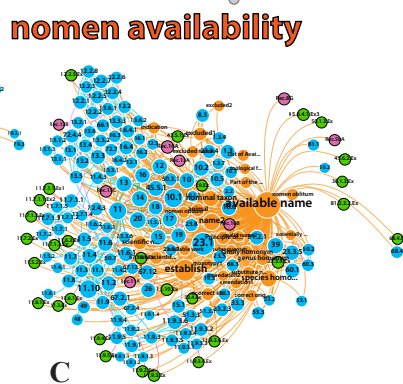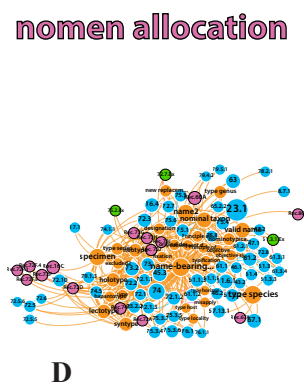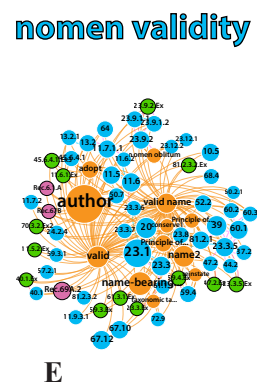

Supplement: Figure S3 — A, the main stages of the nomenclatural process are mapped with different colors in the network, by using a central term and its 1tˆ Degree connections (Ego networks in Gephi). Each subset is separated in its own Force Atlas 2 network. B, the nomenclatural process starts with the publication of a name (bluish green). C, publication creates available names (vermillon). D, these names are allocated to taxa with name-bearing types (reddish purple). E, then, names enter nomenclature to compete for validity (sky blue). [file peerj-07-8127-s006.pdf]
